# Metabolic syndrome parameters' variability and stroke incidence in hypertensive patients: evidence from a functional community cohort

**DOI:** 10.1186/s12933-024-02282-3

**Published:** 2024-06-15

**Authors:** Qitong Liu, Shouling Wu, Jinang Shao, Yang Liu, Yanqiu Lu, Hao Wu, Yan Tian, Yanan Ma, Jingli Gao

**Affiliations:** 1 Key Laboratory of Environmental Stress and Chronic Disease Control & Prevention, Ministry of Education, China Medical University; Department of Biostatistics and Epidemiology, School of Public Health, China Medical University, Shenyang, 110122 Liaoning China; 2 Department of Cardiology, Kailuan General Hospital, No. 57 Xinghua East Road, Heibei 063000 Tangshan, China; 3 Department of Biostatistics and Epidemiology, School of Public Health, North China University of Science and Technology, Tangshan, Hebei China; 4 Department of Intensive Care Unit, Kailuan General Hospital, North China University of Science and Technology, Hebei 063000 Tangshan, China

**Keywords:** Metabolic syndrome parameters, Variability, Stroke, Hypertension, Hypertension complications

## Abstract

**Background:**

Stroke is a common complication of hypertension, but the predictive value of metabolic syndrome parameters' variability on stroke risk in individuals with hypertension remains unclear. Therefore, our objective was to investigate the relationship between metabolic syndrome parameters' variability and the risk of total stroke and its subtypes in hypertensive patients.

**Methods:**

This prospective cohort study included 17,789 individuals with hypertension from the Kailuan study since 2006. Metabolic syndrome parameters, including waist circumference (WC), fasting blood glucose (FBG), systolic blood pressure (SBP), high-density lipoprotein cholesterol (HDL-C), and triglyceride (TG), were collected at three follow-up visits in the 2006, 2008, and 2010 surveys. We assess the variability utilizing the coefficient of variation (CV), standard deviation (SD), average real variation (ARV), and variability independent of the mean (VIM), with CV initially assessed. Participants were categorized based on the number of high-variability metabolic syndrome parameters (0, 1, 2, ≥ 3). Stroke cases were identified by reviewing medical records. The associations between variability in metabolic syndrome parameters and the risk of total stroke and its subtypes were analyzed using Cox proportional hazard regression models.

**Results:**

During a median follow-up of 9.32 years, 1223 cases of stroke were recorded. Participants with ≥ 3 high-variability metabolic syndrome parameters had an increased risk of total stroke (HR: 1.29, 95%CI 1.09–1.52), as well as an increased risk of ischemic stroke (HR: 1.31, 95%CI 1.05–1.63) compared to those without high-variability parameters. The study also examined variability in each metabolic syndrome parameter, and significant associations with an increased risk of total stroke were observed for variability in SBP (HR: 1.24, 95%CI 1.05–1.46) and HDL-C (HR: 1.34, 95%CI 1.09–1.64).

**Conclusions:**

Long-term fluctuations in metabolic syndrome parameters significantly increase the risk of total stroke, especially ischemic stroke. Maintaining low variability in metabolic syndrome parameters could benefit health, and hypertensive individuals must be regularly monitored.

**Supplementary Information:**

The online version contains supplementary material available at 10.1186/s12933-024-02282-3.

## Introduction

Stroke still ranks as the second-leading global cause in terms of death, with about 6.55 million deaths (11.6%) owing to stroke [1]. In China, almost 17.8 million people experienced a stroke, of which 2.3 million died owing to stroke [2]. Among the general population, the prevalence of stroke is 2.6%, but it surged to 44.8% in individuals with hypertension [3]. Hypertension is affecting about one-third of adults worldwide [4], and it is worth noting that the incidence risk is still increasing in China [5]. The need for identifying modifiable factors, particularly those that can avert the devastating consequences of stroke in hypertensive populations, is indisputable.

Metabolic parameters as static variables have been shown to play roles in increased stroke incidence [6–9]. Nevertheless, the metabolic parameters are susceptible to fluctuation over time, affecting health outcomes [10], the static indices alone may not suffice to assess and elucidate the long-term variability. In recent studies, long-term fluctuation in various metabolic parameters is linked to stroke incidence [11–21], irrespective of the initial baseline and average levels. Waist circumference (WC), fasting blood glucose (FBG), systolic blood pressure (SBP), high-density lipoprotein cholesterol (HDL-C), and triglyceride (TG) are interconnected risk factors collection that constitute the metabolic syndrome. Researchers have highlighted the role of combinations in metabolic syndrome components in adverse health outcomes, particularly concerning stroke and its subtypes [22]. Given the potential for their interaction manner [23], combined variability in metabolic syndrome components might exert a more pronounced effect on health. However, up to date, studies have concentrated on examining the impact of fluctuations in metabolic indicators on various unfavorable outcomes, including diabetes [24], cancer [25], arterial stiffness [26], cardiovascular diseases [27–30], and increased mortality [27]. There is a lack of research on how the individual and combined variability of metabolic syndrome parameters affect the risk of total stroke and its subtypes among individuals with hypertension.

We aim to address these gaps, utilizing a population-based database from the Kailuan study to determine the consequence of metabolic syndrome parameters' fluctuation on subsequent stroke risk among hypertensive patients. We will also demonstrate the association between long-term variability in metabolic parameters with ischemic and hemorrhagic stroke.

## Methods

### Study participants

Details of the design, methodology, and procedure were specified previously [31, 32]. In brief, we utilized data from the Kailuan study, a large community-based cohort study performed in northern China, with the participants being active and retired employees. From June 2006 to October 2007, the study was initially carried out, 101,510 employees and retirees (81,110 men and 20,400 women) were recruited from the Kailuan General Hospital and its 11 subsidiary hospitals. The subsequent surveys were issued biennially, with a total of eight visits for comprehensive examinations that included standardized questionnaire assessments, anthropometric measurements, clinical examinations, and laboratory tests up to December 31, 2020.

**Fig. 1:**
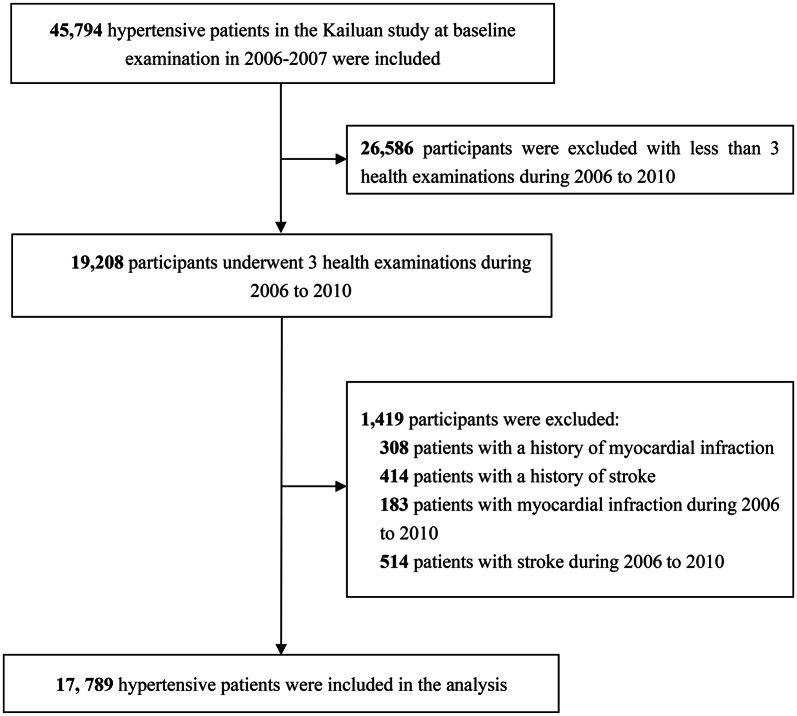
Flow diagram for participants include in the study

For this study, 45,794 hypertensive participants in the 2006 survey were initially selected. A total of 26,586 individuals who were missing complete repeated measurements of metabolic syndrome parameters were excluded, comprising 23,185 participants without stroke or myocardial infarction. Additionally, participants were excluded if they had a history of stroke or myocardial infarction; ultimately, a longitudinal analysis was conducted on a total of 17,789 individuals to examine variability in metabolic syndrome parameters and incident stroke. The flowchart of participant selection is available in Fig. 1.

The research was performed following the guidelines of the World Medical Association Declaration of Helsinki and received ethical approval from the Ethics Committee of the Kailuan General Hospital. Written consent was procured from all participants or their legal representatives before every survey enrollment.

### Data collection

Information on demographic characteristics (age, gender, and education level), sociodemographic parameters (household income and occupation), lifestyle characteristics (alcohol consumption, smoking status, and physical activity), as well as family history of diseases and medication history (diabetes, stroke, myocardial infarction, and current treatments comprising antihypertensive, lipid-lowering, and antidiabetic drugs) was documented by well-trained physicians or nurses via structured questionnaire interviews. Education level was categorized as elementary school or below, middle school, and high school or above. Household income was categorized as < 1000 or ≥ 1000 per month. Smoking status was divided into never, ever, and current. Alcohol consumption was grouped as never, moderate, and heavy. After the participant sat for 15 min and refrained from smoking or drinking for 30 min, their blood pressure was measured. The measurement was conducted on the right upper arm by trained field workers utilizing a calibrated mercury sphygmomanometer. Three consecutive measuring values of blood pressure were obtained, with 5 min of rest between each measurement. The criteria for diagnosis of hypertension are SBP ≥ 140 mmHg, diastolic blood pressure (DBP) of ≥ 90 mmHg, self-reported specialist‐diagnosis history, or the use of antihypertensive medication [38].

Anthropometric measurements were measured by professionally trained physicians utilizing standardized instruments and protocols. Participants were asked to stand barefoot when measuring height, utilizing a tape rule with 0.1 cm accuracy. Weight was assessed utilizing calibrated platform scales with 0.1 kg accuracy in light clothing.

For venous blood sample detection, individuals fasted for 8–12 h, and blood samples were obtained during the routine examination day and infused into EDTA-vacuum tubes. The plasma was detached from the blood instantly and stored at − 80 °C for 4 h, with a CV of < 2.0% on blind quality control samples. All biochemical indicators were detected on the Hitachi 747 auto-analyzer, with the inter-assay CV being < 10% when using the autoanalyzer. The hexokinase/glucose-6-phosphate dehydrogenase method was utilized to measure FBG levels [33]. Serum TC, TG, low-density lipoprotein cholesterol (LDL-C), and HDL-C concentrations were all gauged using the enzymatic colorimetric method. Serum high-sensitivity C-reactive protein (Hs-CRP) level was gauged by the high-sensitivity particle-enhanced immunonephelometry assay and was log-transformed due to skewed distribution.

### Assessment of variability in the metabolic metrics

The variability of each metabolic metric was established as the intra-individual variability in WC, SBP, FBG, HDL-C, and TG values across three measures in 2006, 2008, and 2010. Variability in metabolic metrics was assessed across four indices: coefficient of variation (CV), standard deviation (SD), average real variability (ARV), and variability independent of the mean (VIM). The CV was determined by dividing the standard deviation (SD) by the mean and was calculated accordingly:

$$SD=\sqrt{\frac{{\sum }_{i-1}^{N}{({x}_{i}-\overline{x})}^{2}}{N-1}},$$

where ${x}_{i}$ represents the ${i}^{th}$ point value of each metabolic parameter, $\overline{x}$ is the mean, and *N* is the records of the metabolic metric number.

ARV refers to the mean value of the complete discrepancies between successive measurements, where N indicates the records of the metabolic metric number and i ranges from 1 to N-1. As such,

$$ARV=\frac{1}{\text{N}-1}\sum_{i-1}^{N-1}\left|{value}_{i+1}-{value}_{i}\right|,\text{ in this study},\text{ N}\hspace{0.17em}=\hspace{0.17em}3$$

Taking WC to exemplify, the VIM is computed first as the SD divided by the mean to the power of x, where x was derived through nonlinear regression analysis of the entire sample utilizing the PROC NLIN procedure in the SAS package[34]. As such,

$$VIM=\frac{\text{k}*\text{SD}(\text{WC})}{{\text{Mean}(\text{WC})}^{x}}, \text{where k}\hspace{0.17em}=\hspace{0.17em}\text{Mean}(\text{Mean}(\text{WC}))^{x}$$

The CV was employed as the initial metric for variability assessment. The variability of each metabolic parameter was categorized based on quartiles, with high variability assigned as the highest quartile(Q4) and the lower three quartiles (Q1-Q3) representing low variability. Individuals were stratified based on the cumulative count of metabolic syndrome parameters exhibiting high variability (WC, SBP, FBG, HDL-C, and TG), with the number ranging from 0 to 5, where 0 indicated the absence of high-variability metabolic syndrome parameters and scores ranging from 1 to 5 represented the presence of varying quantity of high-variability parameters. The participants were categorized into four groups due to the presence of 0, 1, 2, or ≥ 3 high-variability metabolic parameters.

### Outcome ascertainment

The outcome was the first onset of stroke, classified into two main subtypes: hemorrhagic stroke and ischemic stroke, while hemorrhagic stroke encompassed both intracerebral and subarachnoid hemorrhage [35]. Examinations of incident stroke have been previously reported [36, 37]. Briefly, fatal and non-fatal cases were adjudicated from four complementary sources: (1) the Provincial Bureau of Life's Statistics and the Municipal Insurance record; (2) discharge lists from 11 hospitals in the Kailuan Group; (3) death certificates; (4) biennial self-report interviews conducted since 2006 (the cohort baseline). All stroke cases were confirmed based on coding from the ICD-Ninth and Tenth Revision (I60 to I61 for hemorrhagic stroke and I63 for ischemic stroke). The mortality information was obtained through medical records, autopsies, and death certificates, with stroke listed as the primary or contributing cause of death. The sudden onset of focal neurologic symptoms due to vascular disease that lasted over 24 h was utilized to identify nonfatal stroke events and verified by brain computed tomography (CT) and magnetic resonance imaging (MRI). Stroke events were further adjudicated by a panel of three experienced professionals, comprising a cardiologist, a neurologist, and a radiologist, who were unaware of both the study's methodology and the participants' exposure status.

### Statistical analysis

The baseline characteristics were assessed, with continuous variables reported as mean ± SD or median (25–75%) based on distribution, and categorical variables as frequencies with percentages. Comparisons between groups were carried out utilizing the one-way ANOVA and the Mann–Whitney *U* test for normally distributed and skewed data, respectively, whereas categorical variables used the χ2 tests.

The CV was initially employed to evaluate the variability of metabolic syndrome parameters. The robustness of variability was assessed employing SD, ARV, and VIM. Spearman's correlation coefficient was employed to analyze the correlation between variability in WC, SBP, FBG, HDL-C, and TG. The cumulative incidence of total stroke and its subtypes were assessed utilizing Kaplan–Meier curves. Differences between survival curves were evaluated utilizing the log-rank test. Cox proportional hazards regression models were utilized to evaluate hazard ratios (HRs) and 95% confidence intervals (CIs) for the risk of total stroke and its subtypes over the period 2010 to 2020 across the number of high-variability parameters, with 0 high-variability metabolic indicators as the reference group. The Schoenfeld residual analysis was conducted to verify the proportional hazards assumption, and no variables were found to be violated. By dividing the stroke cases per 1000 person-years within the high-variability metabolic syndrome parameter category by the incident cases, stroke incidence rates were calculated. Person-years were determined by duration from the index year to the initial occurrence of the listed outcomes: incident stroke, mortality, or end of follow-up. We constructed three nested models step-by-step. Model 1 was non-adjusted; Model 2 adjusted for age (years) and gender; Model 3 additionally adjusted for education level, occupation, household income, smoking status, alcohol consumption, physical activity, dietary quality, antihypertensive, lipid-lowering, antidiabetic drugs, family history of diabetes, family history of stroke, family history of myocardial infarction, and averaged BMI, Hs-CRP during 2006–2010.

Several sensitivity analyses were conducted. (1) The internal correlation between the variability of metabolic parameters was examined; (2) Additional measurements including SD, ARV, and VIM were employed to determine the parameters variability; (3) Stratified by age (< 45, 45–65, 65–85, ≥ 85), gender (women, men), household income per month (< 1000, ≥ 1000 ¥), education (primary school or below, middle school, high or above), occupation (coalminers, other blue, or white collars), alcohol (never, moderate, heavy), smoking status (never, former, current), dietary quality (favorable, intermediate, unfavorable), physical activity (no, occasional, regular) categories were performed utilizing stratified analysis and interaction testing; (4) A lag analysis, excluding stroke events happening in the initial 2 years, was carried out; (5) Aiming at exploring the confounding effect of medications, participants who used antihypertensive, lipid-lowering, and antidiabetic agents were additionally excluded; (6) Individuals who had cancer or atrial fibrillation during follow-up were excluded; (7) After imputing the missing covariates by chained equations, the results remained consistent with the core results; (8) Our analyses were repeated by a Fine-Gray proportion subdistribution hazards model with death accounting for the competing event; (9) We substituted DBP for SBP to form quartiles of variability in metabolic parameters; (10) The *E*-value utilized to detect unmeasured confounding bias is also calculated.

Statistical analyses were accomplished with STATA V17 (STATA Corp LLC, College Station, TX) and SAS Version 9.4 (SAS Institute, Cary, NC, USA), with a 2-sided *P* < 0.05 considered statistically significant.

## Results

### Characteristics of participants

The demographic characteristics are delineated in Table 1. The present analysis involved 17,789 individuals with a mean age of 52.45 ± 10.92 at baseline. Over a median of 9.32 years follow-up, 1223 total stroke incidents were identified, and 1960 (11.02%) had ≥ 3 high-variability metabolic parameters between 2006 and 2010. In comparison to the group without high-variability parameters, the other groups showed significantly higher levels of SBP, DBP, FBG, HDL-C, LDL-C, TG, and Hs-CRP (*P* < 0.001), while there were no differences in baseline BMI or TC. The comparison of the characteristics between the included (n = 17,789) and not included population (n = 23,185) was presented in Table S21.

**Table 1:** Baseline characteristics of subjects by number of metabolic parameters variability according to the coefficient of variation

Variable	Total	Metabolic parameters variability				*P*-value
		0	1	2	≥ 3	
N	17,789 (100.00)	4403 (24.75)	6867 (38.60)	4559 (25.63)	1960 (11.02)	
Age, years^b^	52.45 ± 10.92	52.15 ± 11.01	52.51 ± 10.92	52.56 ± 10.84	52.70 ± 10.92	0.164
Gender, %^a^						0.090
Women	2963 (16.66)	711 (16.15)	1109 (16.15)	787 (17.26)	356 (18.16)	
Men	14,826 (83.34)	3692 (83.85)	5758 (83.85)	3772 (82.74)	1604 (81.84)	
Household income, %^a^						**0.009**
< 1000	13,137 (74.70)	3335 (76.49)	5060 (74.50)	3333 (73.98)	1409 (73.01)	
≥ 1000	4450 (25.30)	1025 (23.51)	1732 (25.50)	1172 (26.02)	521 (26.99)	
Education level, %^a^						** < 0.001**
Elementary school or below	1670 (9.40)	355 (8.08)	657 (9.58)	460 (10.11)	198 (10.11)	
Middle school	13,008 (73.23)	3151 (71.68)	4988 (72.72)	3377 (74.20)	1492 (76.20)	
High school or above	3086 (17.37)	890 (20.25)	1214 (17.70)	714 (15.69)	268 (13.69)	
Occupation, %^a^						**0.015**
Coal miners	5273 (30.57)	1283 (29.88)	2018 (30.31)	1372 (31.13)	600 (31.76)	
Other blue collars	10,966 (63.58)	2715 (63.23)	4253 (63.89)	2800 (63.54)	1198 (63.42)	
White collars	1008 (5.84)	296 (6.89)	386 (5.80)	235 (5.33)	91 (4.82)	
Alcohol consumption, %^a^						0.913
Never	10,475 (60.74)	2575 (60.02)	4051 (60.74)	2694 (61.27)	1155 (61.11)	
Moderate	594 (3.44)	153 (3.57)	223 (3.34)	155 (3.53)	63 (3.33)	
Heavy	6177 (35.82)	1562 (36.41)	2395 (35.91)	1548 (35.21)	672 (35.56)	
Smoking status, %^a^						**0.032**
Never	11,163 (62.80)	2766 (62.88)	4297 (62.61)	2838 (62.32)	1262 (64.42)	
Ever	835 (4.70)	216 (4.91)	349 (5.09)	207 (4.55)	63 (3.22)	
Current	5777 (32.50)	1417 (32.21)	2217 (32.30)	1509 (33.14)	634 (32.36)	
Dietary quality, %^a^						0.484
Favorable	3230 (18.17)	787 (17.89)	1240 (18.07)	836 (18.35)	367 (18.73)	
Moderate	12,645 (71.15)	3161 (71.87)	4846 (70.63)	3253 (71.42)	1385 (70.70)	
Unfavorable	1898 (10.68)	450 (10.23)	775 (11.30)	466 (10.23)	207 (10.57)	
Physical activity, %^a^						**0.013**
Inactive	5015 (28.21)	1201 (27.30)	1983 (28.89)	1257 (27.60)	574 (29.32)	
Occasional	10,065 (56.62)	2487 (56.54)	3836 (55.89)	2607 (57.23)	1135 (57.97)	
Regular	2696 (15.17)	711 (16.16)	1045 (15.22)	691 (15.17)	249 (12.72)	
Family history of diabetes, %^a^						0.400
No	17,175 (97.01)	4269 (97.35)	6618 (96.88)	4391 (96.80)	1897 (97.13)	
Yes	530 (2.99)	116 (2.65)	213 (3.12)	145 (3.20)	56 (2.87)	
Family history of stroke, %^a^						0.491
No	16,983 (95.47)	4219 (95.82)	6546 (95.33)	4355 (95.53)	1863 (95.05)	
Yes	806 (4.53)	184 (4.18)	321 (4.67)	204 (4.47)	97 (4.95)	
Family history of MI, %^a^						0.086
No	17,426 (97.96)	4296 (97.57)	6726 (97.95)	4474 (98.14)	1930 (98.47)	
Yes	363 (2.04)	107 (2.43)	141 (2.05)	85 (1.86)	30 (1.53)	
Antihypertensive drugs, %^a^						0.162
No	14,108 (79.31)	3488 (79.22)	5444 (79.28)	3586 (78.66)	1590 (81.12)	
Yes	3681 (20.69)	915 (20.78)	1423 (20.72)	973 (21.34)	370 (18.88)	
Lipid-lowering drugs, %^a^						**0.016**
No	17,519 (98.48)	4354 (98.89)	6767 (98.54)	4472 (98.09)	1926 (98.27)	
Yes	270 (1.52)	49 (1.11)	100 (1.46)	87 (1.91)	34 (1.73)	
Antidiabetic drugs, %^a^						** < 0.001**
No	16,998 (95.55)	4304 (97.75)	6601 (96.13)	4294 (94.19)	1799 (91.79)	
Yes	791 (4.45)	99 (2.25)	266 (3.87)	265 (5.81)	161 (8.21)	
WC, cm^b^	89.07 ± 9.56	89.19 ± 8.42	88.82 ± 9.31	89.26 ± 10.28	89.26 ± 10.96	**0.045**
BMI, kg/m^2b^	26.09 ± 3.43	26.14 ± 3.23	26.02 ± 3.42	26.13 ± 3.51	26.11 ± 3.64	0.240
SBP, mmHg^b^	146.07 ± 16.83	143.83 ± 14.66	145.53 ± 16.48	147.34 ± 17.45	150.05 ± 19.93	** < 0.001**
DBP, mmHg^b^	92.70 ± 9.72	92.01 ± 9.06	92.59 ± 9.51	93.09 ± 9.91	93.73 ± 11.25	** < 0.001**
FBG, mmol/L^b^	5.61 ± 1.75	5.34 ± 1.13	5.53 ± 1.55	5.78 ± 2.08	6.12 ± 2.45	** < 0.001**
HDL-C, mmol/L^b^	1.58 ± 0.41	1.54 ± 0.32	1.56 ± 0.38	1.61 ± 0.47	1.66 ± 0.52	** < 0.001**
LDL-C, mmol/L^b^	2.36 ± 1.00	2.39 ± 0.91	2.38 ± 1.00	2.34 ± 1.07	2.28 ± 1.06	** < 0.001**
TG, mmol/L^c^	1.45 (1.05, 2.26)	1.34 (1.01, 1.90)	1.41 (1.03, 2.11)	1.59 (1.11, 2.65)	1.76 (1.14, 3.13)	** < 0.001**
TC, mmol/L^c^	5.00 (4.35, 5.69)	5.01 (4.39, 5.65)	4.98 (4.34, 5.66)	5.01 (4.32, 5.74)	5.01 (4.29, 5.76)	0.313
Hs-CRP, mg/L^c^	0.90 (0.33, 2.70)	0.85 (0.32, 2.31)	0.90 (0.32, 2.67)	0.97 (0.31, 3.00)	1.05 (0.36, 3.10)	** < 0.001**
Variability, %^b^						
WC	6.48 ± 4.49	4.55 ± 2.08	6.22 ± 4.24	7.55 ± 5.04	9.25 ± 5.65	** < 0.001**
SBP	8.35 ± 4.77	6.20 ± 2.72	7.84 ± 4.40	9.65 ± 5.23	11.97 ± 5.45	** < 0.001**
FBG	11.61 ± 9.17	7.58 ± 3.55	10.53 ± 7.86	13.95 ± 10.52	19.03 ± 12.38	** < 0.001**
HDL-C	18.81 ± 12.16	13.46 ± 6.11	17.43 ± 11.07	22.28 ± 13.52	27.53 ± 15.15	** < 0.001**
TG	32.53 ± 21.03	22.63 ± 10.26	29.95 ± 19.04	38.77 ± 23.59	49.32 ± 24.47	** < 0.001**

Data were incomplete for these variables: 1.14% (n = 202), 0.14% (n = 25), 3.05% (n = 542), 3.05% (n = 543), 0.08% (n = 14), 0.09% (n = 16), 0.07% (n = 13), 0.47% (n = 84), 0.06% (n = 10), 0.17% (n = 30) of participants had missing data for Household income, Education level, Occupation, Alcohol consumption, Smoking status, Dietary quality, Physical activity, Family history of diabetes, BMI, and LDL-C, respectively. Other variables included in the analyses did not have missing data. Values highlighted in bold indicate statistical significance.

*MI* myocardial infarction, *WC* waist circumference, *BMI* body mass index, *SBP* systolic blood pressure, *DBP* diastolic blood pressure, *FBG* fasting blood glucose, *HDL-C* high-density lipoprotein cholesterol, *LDL-C* low-density lipoprotein cholesterol, *TG* triglycerides, *TC* total cholesterol, *Hs-CRP* high-sensitivity C-reactive protein

^a^These variables were examined by χ2 tests with H0

^b^These variables were examined by 1-way ANOVA with H0

^c^These variables were examined by Mann–Whitney U tests with H0. H0: There was no statistical difference between the tested variables in the number of metabolic parameters variability (*P* > 0.05)

### Risk of stroke according to high-variability parameters number

**Fig. 2:**
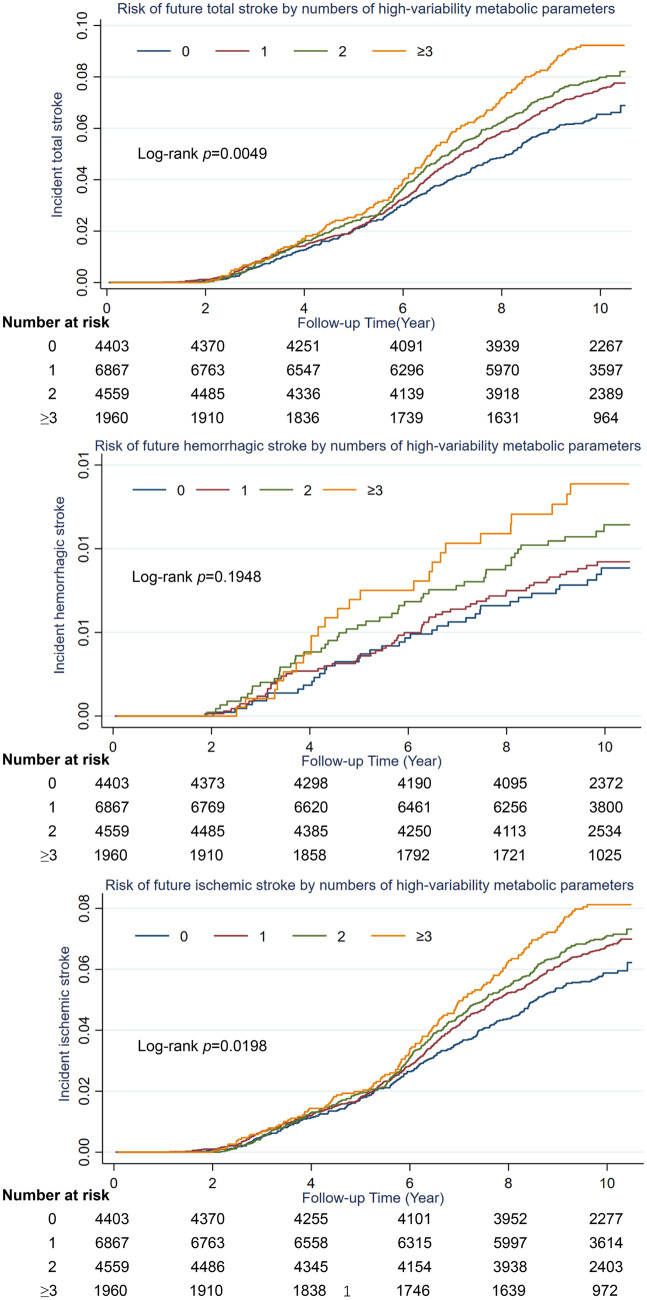
Kaplan–Meier curves showing the cumulative incidence of total stroke, hemorrhage stroke, and ischemic stroke according to numbers of high-variability metabolic syndrome parameters

The high-variability parameters' number revealed the combined effect of all parameter variability. After adjusting for all covariates, compared with individuals who had 0 high-variability parameters, participants who had three or more of all the five high-variability metabolic parameters assessed by the CV exhibited an elevated risk of total stroke risk (HR: 1.34, 95%CI 1.09–1.64) and ischemic stroke risk (HR: 1.31, 95%CI 1.05–1.63). There was no significant association found between the number of high-variability parameters and hemorrhagic stroke risk. However, the incidence rates of hemorrhagic stroke escalated in correlation with the quantity of metabolic syndrome parameters exhibiting high variability: 0.850, 0.902, 1.110, and 1.367 per 1000 person-year for 0, 1, 2, ≥ 3 high-variability metabolic parameters **(**Table 2**)**. The risk of total stroke and its subtypes was further estimated by calculating the variability of metabolic syndrome parameters utilizing SD, ARV, and VIM (Tables S3–S5). Meanwhile, the Kaplan–Meier curves exhibited similar results (log-rank test, *P* < 0.001) in Fig. 2. Besides, multiple sensitivity analyses are presented in Tables S6–S20.

**Table 2:** Hazard ratios (HRs) and 95% confidence intervals (95%CIs) of incidence stroke of metabolic parameter variability according to the coefficient of variation

Outcome	Variable	Case/Total, N	Follow-up duration (Person-years)	Incidence rate, per 1000 person-years	HR (95%CI)		
					Model 1	Model 2	Model 3
Total stroke	0	265/4403	41,544.70	6.379	1.0 (Reference)	1.0 (Reference)	1.0 (Reference)
	1	471/6867	64,021.07	7.357	1.16 (0.99, 1.35)	1.15 (0.99, 1.34)	1.15 (0.99, 1.35)
	2	328/4559	42,294.10	7.755	**1.22 (1.04, 1.44)**	**1.22 (1.04, 1.44)**	1.18 (1.00, 1.40)
	≥ 3	159/1960	17,845.74	8.910	**1.41 (1.16, 1.72)**	**1.42 (1.16, 1.73)**	**1.34 (1.09, 1.64)**
	*P* for trend				** < 0.001**	** < 0.001**	**0.005**
	1 point increment				**1.11 (1.05, 1.18)**	**1.11 (1.05, 1.18)**	**1.09 (1.02, 1.16)**
Hemorrhagic stroke							
	0	36/4403	42,347.88	0.850	1.0 (Reference)	1.0 (Reference)	1.0 (Reference)
	1	59/6867	65,429.17	0.902	1.06 (0.70, 1.61)	1.06 (0.70, 1.60)	1.07 (0.69, 1.65)
	2	48/4559	43,249.17	1.110	1.31 (0.85, 2.02)	1.31 (0.85, 2.02)	1.17 (0.74, 1.86)
	≥ 3	25/1960	18,287.41	1.367	1.62 (0.97, 2.70)	1.62 (0.97, 2.70)	1.56 (0.91, 2.67)
	*P* for trend				**0.035**	**0.034**	0.095
	1 point increment				**1.18 (1.01, 1.38)**	**1.18 (1.01, 1.38)**	1.14 (0.97, 1.35)
Ischemic stroke							
	0	239/4403	41,620.36	5.742	1.0 (Reference)	1.0 (Reference)	1.0 (Reference)
	1	424/6867	64,161.28	6.608	1.15 (0.99, 1.35)	1.15 (0.98, 1.35)	1.15 (0.98, 1.36)
	2	292/4559	42,407.76	6.886	**1.21 (1.02, 1.43)**	**1.21 (1.02, 1.43)**	1.18 (0.99, 1.41)
	≥ 3	140/1960	17,899.29	7.822	**1.38 (1.12, 1.70)**	**1.38 (1.12, 1.70)**	**1.31 (1.05, 1.63)**
	*P* for trend				**0.002**	**0.002**	**0.013**
	1 point increment				**1.10 (1.03, 1.17)**	**1.10 (1.04, 1.17)**	**1.08 (1.02, 1.16)**

Model 1 was non-adjusted;

Model 2 was adjusted for age and gender

Model 3 was further adjusted for education level (elementary school or below; middle school; high school or above), occupation (coal miners; other blue collars; white collars), household income (< 1000 ¥; ≥ 1000 ¥), smoking status (never; ever; current), alcohol consumption (never; moderate; heavy), physical activity (inactive; occasional; regular), dietary quality (favorable; moderate; unfavorable), antihypertensive drugs (yes or no), lipid-lowering drugs (yes or no), antidiabetic drugs (yes or no), family history of diabetes (yes or no), family history of stroke (yes or no), family history of myocardial infarction (yes or no), and mean of body mass index, high-sensitivity C-reactive protein during 2006 to 2010. Values highlighted in bold indicate statistical significance.

### Risk of stroke according to each parameter variability

The individuals were stratified based on the quartiles of the CV of each metabolic syndrome parameter, ranging from lowest to highest variability. Compared to the lowest quartile of SBP variability assessed by the CV, the highest quartile was observed with greater total stroke risk (HR: 1.24, 95%CI 1.05–1.46). Similarly, individuals with higher variability in HDL-C assessed by the CV exhibited a pronounced increase in total stroke risk compared to those in the lowest quartile (HR: 1.20, 95%CI 1.02–1.42 in the third quartile group; HR: 1.29, 95%CI 1.09–1.52 in the fourth quartile group). The high variability group (Q4) of FBG exhibited a higher incidence of total stroke rate compared to the reference group (Q1). No significant associations were observed between total stroke risk and variability in WC and TG levels (Table 3).

**Table 3:** Hazard ratios (HRs) and 95% confidence intervals (95%CIs) of total stroke by quartiles of metabolic parameter variability according to the coefficient of variation

Variable	Case/Total, N	Follow-up duration (Person-years)	Incidence rate, per 1000 person-years	HR (95%CI)		
				Model 1	Model 2	Model 3
Waist circumstance						
Q1	309/4448	41,933.47	7.367	1.0 (Reference)	1.0 (Reference)	1.0 (Reference)
Q2	293/4450	41,651.91	7.035	0.96 (0.81, 1.12)	0.96 (0.82, 1.13)	0.95 (0.81, 1.12)
Q3	317/4445	41,290.31	7.677	1.04 (0.89, 1.22)	1.03 (0.88, 1.20)	1.03 (0.87, 1.21)
Q4	304/4446	40,829.91	7.446	1.01 (0.87, 1.19)	0.97 (0.82, 1.13)	0.95 (0.81, 1.13)
*P* for trend				0.650	0.817	0.791
1 point increment				1.01 (0.96, 1.07)	1.00 (0.95, 1.05)	0.99 (0.94, 1.05)
Systolic blood pressure						
Q1	287/4448	42,032.15	6.828	1.0 (Reference)	1.0 (Reference)	1.0 (Reference)
Q2	298/4534	42,408.35	7.027	1.03 (0.88, 1.21)	1.02 (0.87, 1.20)	1.02 (0.86, 1.20)
Q3	287/4360	40,654.39	7.060	1.04 (0.88, 1.22)	1.01 (0.86, 1.20)	1.01 (0.85, 1.20)
Q4	351/4447	40,610.70	8.643	**1.28 (1.09, 1.49)**	**1.24 (1.06, 1.45)**	**1.24 (1.05, 1.46)**
*P* for trend				**0.001**	**0.005**	**0.005**
1 point increment				**1.08 (1.03, 1.14)**	**1.07 (1.02, 1.12)**	**1.07 (1.01, 1.13)**
Fasting blood glucose						
Q1	271/4448	41,931.71	6.463	1.0 (Reference)	1.0 (Reference)	1.0 (Reference)
Q2	316/4447	41,496.39	7.615	**1.18 (1.005, 1.39)**	1.14 (0.97, 1.34)	1.14 (0.96, 1.34)
Q3	296/4447	41,429.19	7.145	1.11 (0.94, 1.31)	1.06 (0.90, 1.25)	1.06 (0.89, 1.25)
Q4	340/4447	40,848.30	8.324	**1.30 (1.11, 1.52)**	**1.25 (1.07, 1.47)**	1.13 (0.95, 1.34)
*P* for trend				**0.004**	**0.013**	0.316
1 point increment				**1.07 (1.02, 1.13)**	**1.06 (1.01, 1.12)**	1.03 (0.97, 1.08)
High-density lipoprotein						
Q1	286/4448	41,374.63	6.912	1.0 (Reference)	1.0 (Reference)	1.0 (Reference)
Q2	288/4447	41,638.66	6.917	1.00 (0.85, 1.18)	1.04 (0.88, 1.22)	1.10 (0.92, 1.30)
Q3	308/4447	41,496.77	7.422	1.08 (0.92, 1.26)	1.13 (0.96, 1.33)	**1.20 (1.02, 1.42)**
Q4	341/4447	41,195.55	8.278	**1.20 (1.03, 1.41)**	**1.23 (1.05, 1.44)**	**1.29 (1.09, 1.52)**
*P* for trend				**0.009**	**0.004**	**0.001**
1 point increment				**1.07 (1.01, 1.12)**	**1.07 (1.02, 1.13)**	**1.09 (1.03, 1.15)**
Triglycerides						
Q1	317/4448	41,287.65	7.678	1.0 (Reference)	1.0 (Reference)	1.0 (Reference)
Q2	314/4447	41,150.86	7.631	1.00 (0.85, 1.16)	1.00 (0.86, 1.17)	0.98 (0.84, 1.16)
Q3	291/4447	41,360.72	7.036	0.92 (0.78, 1.07)	0.95 (0.81, 1.11)	0.93 (0.79, 1.09)
Q4	301/4447	41,906.38	7.183	0.93 (0.80, 1.09)	1.03 (0.88, 1.21)	1.01 (0.86, 1.20)
*P* for trend				0.306	0.798	0.879
1 point increment				0.97 (0.92, 1.02)	1.00 (0.95, 1.05)	1.00 (0.95, 1.05)

Model 1 was non-adjusted

Model 2 was adjusted for age and gender

Model 3 was further adjusted for education level (elementary school or below; middle school; high school or above), occupation (coal miners; other blue collars; white collars), household income (< 1000 ¥; ≥ 1000 ¥), smoking status (never; ever; current), alcohol consumption (never; moderate; heavy), physical activity (inactive; occasional; regular), dietary quality (favorable; moderate; unfavorable), antihypertensive drugs (yes or no), lipid-lowering drugs (yes or no), antidiabetic drugs (yes or no), family history of diabetes (yes or no), family history of stroke (yes or no), family history of myocardial infarction (yes or no), and mean of body mass index, high-sensitivity C-reactive protein during 2006 to 2010. Values highlighted in bold indicate statistical significance.

### Subgroup analysis

The results of subgroup analyses are presented in Table S2. Generally, there is a notable interaction between the quantity of high-variability parameters with age and gender, with the effect of an increased quantity of metabolic parameters exhibiting high variability being more pronounced in young adults aged < 45 and men (*P* for interaction < 0.05).

## Discussion

The study conducted a prospective analysis to examine the effect of metabolic syndrome parameters' variability on the incidence of total stroke and its subtypes in hypertensive patients. In comparison to patients with 0 high-variability parameters, those patients who had ≥ 3 exhibited a significantly elevated risk of total stroke and ischemic stroke. The variability of SBP and HDL-C among parameters exhibits a pronounced relationship with total stroke risk.

Our findings demonstrate that an increase in the quantity of high-variability metabolic syndrome indicators elevates the risk of total stroke and ischemic stroke, suggesting a potential cumulative association between parameter variability and the incidence of total stroke and ischemic stroke. There is no evidence regarding the effect of metabolic syndrome parameters' variability on total stroke and its subtypes, with most studies focusing on unfavorable health outcomes such as diabetes [24], cancer [25], arterial stiffness [26], cardiovascular diseases [27–30], and increased mortality [27]. Researchers in China discovered that the number of high-variability parameters including total cholesterol (TC), uric acid (UA), visceral adiposity index (VAI), BMI, and SBP had implications for diabetes [24]. Another study conducted in Korea showed that high variability in SBP, body weight, FBG, and TC was linked to an increased risk of lung cancer [25]. Additionally, a population-based study suggested that the high number of variabilities in WC, FBG, SBP, TG, and HDL-C elevated cardiovascular disease risk [26]. The association was also noted among diabetic patients, with the metabolic parameters' variability under evaluation being SBP, FBG, TC, and TG [28]. One study examined the association between the variability of metabolic parameters, comprising SBP, BMI, FBG, and TC, and mortality among individuals in a Chinese population [27]. Participants who exhibited high variability in FBG, SBP, TC, and BMI were found to be at increased risk for developing atrial fibrillation [29]. Significant correlations were observed between high-variability of SBP, BMI, FBG, and TC and the development of new-onset heart failure in a synergistic manner [30]. Previous studies have indicated that individuals with diabetes and cerebral ischemia are at a heightened risk for stroke recurrence, with in-hospital mortality rates as high as 14.9% in women, and that the management of blood pressure and glycemic can effectively reduce the risk of early death [39]. Previous research primarily focused on healthy and diabetic populations, whereas few looked at the risk of stroke in hypertensive patients. Additionally, prior research has focused on the variability of different metabolic parameters in the general population or among patients, without considering how the variability of metabolic syndrome parameters, encompassing WC, SBP, FBG, HDL-C, and TG relates to the risk of stroke and its subtypes. The precise impact of metabolic syndrome parameter variability on stroke outcomes in hypertensive patients remains uncertain. Our current research is the pioneering study to describe the long-term variability in metabolic syndrome parameters among participants with hypertension. Additionally, this research serves as the initial exploration into the prognostic significance of fluctuations in the metabolic syndrome indicators in predicting the occurrence of total stroke and its subtypes in hypertensive participants within a single cohort.

Our study suggested that SBP and HDL-C variability were superior to other parameters in predicting the total stroke risk. The impact between fluctuations in blood pressure and stroke incidence has been examined across distinct countries [15–18], with our findings corroborating existing research. Previous investigations exploring lipid variability on stroke are limited, suggesting that variability in HDL-C levels may increase the risk of stroke and ischemic stroke, while no significant association was found for TG levels [19–21]. However, there is a 37% increase in mortality risk for individuals with high TG variability [20], suggesting a potential biological detriment to health with Tg variability. The impact of body weight fluctuation on stroke risk remains unclear. One study found that individuals exhibiting the greatest variability in BMI were associated with a 14% increased likelihood of stroke [27], while another study with a large sample size did not observe a significant relationship [13]. The discrepancy between these studies may be ascribed to distinct measurement of BMI variability. Yeong Min Lim et al. used average absolute difference (ASV) from biennial measurements to assess the variability of BMI, rather than categorizing individuals based on quartile values over three successive years of recording. To date, there exist a paucity of information regarding the variability of WC about the risk of stroke. Our findings did not reveal a substantial correlation between WC variability and heightened risk of stroke; it is possible that being overweight or even obese in elderly individuals may confer a protective effect on health [40]. The enduring fluctuation in glucose levels has garnered increasing attention, and earlier studies corroborate our findings regarding variability in FBG [11, 12]. There is an exact mechanism by which the fluctuations in glucose levels adversely affect endothelial function and oxidative stress compared to average glucose levels [41], ultimately exacerbating the progression of the atherosclerotic process, a crucial factor in the development of stroke [42].

There are several strengths to the study. First, we utilized a sizable, community-based, longitudinal cohort, with participants being followed for an average of 9.32 years. Second, this study presents the first investigation into the long-term variability of metabolic syndrome parameters about total stroke and its subtypes, accounting for both individual and combined effects of these parameters. Third, we conducted diverse variability measures and sensitivity analyses to robust the validity of our results.

However, there are several limitations to take into account. Despite employing a cohort design, the inherent nature of observational research prevented confirmation of a causal relationship between metabolic syndrome parameter variability and stroke. Hence, individuals with previous myocardial infarction and stroke, as well as those who experienced these events between 2006 and 2010, were excluded from the primary analyses. Additionally, multiple sensitivity analyses were conducted, excluding participants with outcome events in the previous 2 years, atrial fibrillation, or cancer, yielding similar results to the primary analyses. Second, while adjusting for all available potential risk factors, residual confounding remains a possibility as certain proven risk factors, such as sleep health, were not included in the data collection process. Third, the assessment of metabolic syndrome parameters at three points may lead to underestimates of variability. Fourth, the study was drawn from the Kailuan community, which is predominantly composed of male coal miners, potentially introducing selection bias. Subgroup analysis was conducted to assess gender disparities, which did not reveal any significant differences. Fifth, our research excluded individuals with missing metabolic syndrome parameters to ensure the completeness of parameters from three follow-up visits, but this may cause selection bias. Therefore, it is imperative to prioritize the annual completeness of metabolic parameters in future research efforts. Finally, our dataset is deficient in detailed classification of stroke subtypes, such as in distinguishing between lacunar and non-lacunar infarcts [43]. Given the substantial differences in pathophysiology, prognosis, and clinical characteristics between these subtypes, it is imperative to conduct a comprehensive investigation into the effects of the metabolic syndrome parameters' variability on specific stroke subtypes. Future studies should prioritize the examination of how variabilities in metabolic parameters influence the risk factors associated with specific stroke subtypes.

In summary, our research investigated the relationship between long-term fluctuations in metabolic syndrome parameters and the risk of total stroke and its subtypes among individuals with hypertension. Recommendations for preventing the development of stroke comorbidities in hypertensive patients are provided for the variability of metabolic parameter indicators. Subsequent research should extrapolate the analysis to the general population and focus on detailed subtypes of stroke through targeted and comprehensive analyses.

## Conclusion

Combinations of variabilities in metabolic syndrome parameters hold predictive value for total stroke and its subtypes in hypertensive patients. A greater number of high-variability metabolic syndrome parameters was positively correlated with elevated risk of total stroke and ischemic stroke. Variabilities in SBP and HDL-C were specifically linked to total stroke risk. These results emphasize the necessity of monitoring long-term fluctuations in metabolic syndrome parameters for health and underscore the need for implementing appropriate measures to mitigate stroke risk.

### Supplementary Information

**Supplementary material file 1.**

## Data Availability

All data-sharing and collaboration requests should be directed to the corresponding author.

## Abbreviations

FBG: Fasting blood glucose
BMI: Body mass index
WC: Waist circumference
SBP: Systolic blood pressure
HDL-C: High-density lipoprotein cholesterol
LDL-C: Low-density lipoprotein cholesterol
TG: Triglycerides
TC: Total cholesterol
UA: Uric acid
VAI: Visceral adiposity index
NHIS: National Health Insurance Service
DBP: Diastolic blood pressure
EDTA: Ethylene diamine tetra-acetic acid
Hs-CRP: High-sensitivity C-reactive protein
eGFR: Estimated glomerular filtration rate
SD: Standard deviation
CV: Coefficient of variation
ARV: Average real variability
VIM: Variability independent of the mean
CT: Computed tomography
MRI: Magnetic resonance
ANOVA: Analysis of variance
HR: Hazard ratio
CI: Confidence interval
ASV: Average absolute difference
