# Supplementary material for: Metabolic syndrome parameters' variability and stroke incidence in hypertensive patients: evidence from a functional community cohort
Source: Cardiovasc Diabetol. 2024 Jun 15;23:203. doi: 10.1186/s12933-024-02282-3 (PMC11180400; doi:10.1186/s12933-024-02282-3)
Supplement: Supplementary file 1 — Supplementary material file 1. [file 12933_2024_2282_MOESM1_ESM.docx]

**Table S1a** Correlation coefficient for the CV of variability of metabolic syndrome parameters.

**Table S1b** Correlation coefficient for the SD of variability of metabolic syndrome parameters.

**Table S1c** Correlation coefficient for the ARV of variability of metabolic syndrome parameters.

**Table S1d** Correlation coefficient for the VIM of variability of metabolic syndrome parameters.

**Table S2** Stratified and interaction analyses of metabolic syndrome parameters variability and incidence of total stroke.

**Table S3** Hazard ratios (HRs) and 95% confidence intervals (95%CIs) of incident stroke by quartiles of metabolic parameters variability according to standard deviation.

**Table S4** Hazard ratios (HRs) and 95% confidence intervals (95%CIs) of incident stroke by quartiles of metabolic parameters variability according to average real variability.

**Table S5** Hazard ratios (HRs) and 95% confidence intervals (95%CIs) of incident stroke by quartiles of metabolic parameters variability according to variability independent of the mean.

**Table S6** Sensitivity analysis of excluding participants with the use of anti-hypertensive drugs (n=3681).

**Table S7** Sensitivity analysis of excluding participants with the use of antidiabetic drugs (n=791).

**Table S8** Sensitivity analysis of excluding participants with the use of lipid-lowering drugs (n=270).

**Table S9** Sensitivity analysis of excluding participants who had the incidence outcome within the first 2 years.

**Table S10** Sensitivity analysis of excluding participants who developed atrial fibrillation.

**Table S11** Sensitivity analysis of excluding participant who developed cancer during 2006 to 2010.

**Table S12** Sensitivity analysis of DBP by SBP formed the metabolic syndrome parameter variability.

**Table S13** Metabolic syndrome parameters’ variability with risk of stroke among patients after multiple imputation of missing data.

**Table S14** Associations of variability with risk of total stroke: competing risk analysis.

**Table S15** The E-value for metabolic syndrome parameters’ variability with risk of stroke among patients with hypertension.

**Table S16** Hazard ratios (HRs) and 95% confidence intervals (95%CIs) of incidence stroke of metabolic parameter variability according to the CV after adjusted for metabolic parameters.

**Table S17** Hazard ratios (HRs) and 95% confidence intervals (95%CIs) of incidence stroke of metabolic parameter variability according to the SD after adjusted for metabolic parameters.

**Table S18** Hazard ratios (HRs) and 95% confidence intervals (95%CIs) of incidence stroke of metabolic parameter variability according to the ARV after adjusted for metabolic parameters.

**Table S19** Hazard ratios (HRs) and 95% confidence intervals (95%CIs) of incidence stroke of metabolic parameter variability according to the VIM after adjusted for metabolic parameters.

**Table S20** Hazard ratios (HRs) and 95% confidence intervals (95%CIs) of total stroke by quartiles of metabolic parameter variability according to the CV after adjusted for metabolic parameters.

**Table S21** Baseline characteristics between participants who were excluded and included.

**Table S1a** Correlation coefficient for the CV of variability of metabolic syndrome parameters.

| ***r*** | **CV of WC** | **CV of SBP** | **CV of FBG** | **CV of HDL** | **CV of TG** |
| --- | --- | --- | --- | --- | --- |
| **CV of WC** | 1.0000 | - | - | - | - |
| **CV of SBP** | **0.0290** | 1.0000 | - | - | - |
| **CV of FBG** | 0.0136 | **0.0332** | 1.0000 | - | - |
| **CV of HDL** | **-0.0326** | **0.0253** | **0.0425** | 1.0000 | - |
| **CV of TG** | 0.0074 | **0.0154** | **0.0665** | **0.0598** | 1.0000 |

***r*** = Pearson correlation coefficient. Pearson’s correlation coefficient was employed to determine the relationship between parameters. Parameters with a ***P***-value less than 0.05 are indicated in bold, indicating a significant correlation between the two metabolic parameters.

Abbreviations: CV, coefficient of variability; SD, standard deviation; ARV, average real variability; VIM, variability independent of the mean;

**Table S1b** Correlation coefficient for the SD of variability of metabolic syndrome parameters.

| ***r*** | **SD of WC** | **SD of SBP** | **SD of FBG** | **SD of HDL** | **SD of TG** |
| --- | --- | --- | --- | --- | --- |
| **SD of WC** | 1.0000 | - | - | - | - |
| **SD of SBP** | **0.0324** | 1.0000 | - | - | - |
| **SD of FBG** | **0.0216** | **0.0329** | 1.0000 | - | - |
| **SD of HDL** | **-0.0235** | **0.0181** | 0.0123 | 1.0000 | - |
| **SD of TG** | -0.0055 | 0.0044 | **0.0685** | **0.0610** | 1.0000 |

**Table S1c** Correlation coefficient for the ARV of variability of metabolic syndrome parameters.

| ***r*** | **ARV of WC** | **ARV of SBP** | **ARV of FBG** | **ARV of HDL** | **ARV of TG** |
| --- | --- | --- | --- | --- | --- |
| **ARV of WC** | 1.0000 | - | - | - | - |
| **ARV of SBP** | **0.0305** | 1.0000 | - | - | - |
| **ARV of FBG** | 0.0144 | **0.0228** | 1.0000 | - | - |
| **ARV of HDL** | **-0.0169** | 0.0101 | 0.0100 | 1.0000 | - |
| **ARV of TG** | -0.0038 | 0.0013 | **0.0656** | **0.0438** | 1.0000 |

**Table S1d** Correlation coefficient for the VIM of variability of metabolic syndrome parameters.

| ***r*** | **VIM of WC** | **VIM of SBP** | **VIM of FBG** | **VIM of HDL** | **VIM of TG** |
| --- | --- | --- | --- | --- | --- |
| **VIM of WC** | 1.0000 | - | - | - | - |
| **VIM of SBP** | **0.0269** | 1.0000 | - | - | - |
| **VIM of FBG** | 0.0119 | **0.0275** | 1.0000 | - | - |
| **VIM of HDL** | **-0.0329** | **0.0213** | **0.0356** | 1.0000 | - |
| **VIM of TG** | **0.0381** | **0.0256** | **0.0416** | -0.0116 | 1.0000 |

**Table S2** Stratified and interaction analyses of metabolic syndrome parameters variability and incidence of total stroke.

| **Subgroup** | **Stroke, HR (95%CI)** | | | | ***P*-interaction** |
| --- | --- | --- | --- | --- | --- |
|  | **0 (N=4403)** | **1 (N=6867)** | **2 (N=4559)** | **≥3 (N=1960)** |  |
| **Age, years** |  |  |  |  | **0.039** |
| <45 (n=4655) | 1.0 (Reference) | 1.04 (0.69, 1.57) | **1.69 (1.13, 2.53)** | **2.08 (1.29, 3.34)** |  |
| 45-65 (n=10657) | 1.0 (Reference) | 1.21 (1.00, 1.47) | 1.12 (0.91, 1.39) | 1.22 (0.94, 1.59) |  |
| 65-85 (n=2460) | 1.0 (Reference) | 1.04 (0.73, 1.48) | 0.98 (0.66, 1.45) | 1.25 (0.78, 2.00) |  |
| ≥85 (n=17) | 1.0 (Reference) | — | — | — |  |
| **Age, years** |  |  |  |  | **0.031** |
| <45 (n=4655) | 1.0 (Reference) | 1.04 (0.69, 1.57) | **1.69 (1.13, 2.53)** | **2.08 (1.29, 3.34)** |  |
| 45-65 (n=10657) | 1.0 (Reference) | 1.21 (1.00, 1.47) | 1.12 (0.91, 1.39) | 1.22 (0.94, 1.59) |  |
| 65-80 (n=2386) | 1.0 (Reference) | 1.07 (0.75, 1.52) | 1.01 (0.69, 1.50) | 1.28 (0.80, 2.05) |  |
| ≥80 (n=91) | 1.0 (Reference) | — | — | — |  |
| **Age, years** |  |  |  |  | **0.035** |
| <45 (n=4655) | 1.0 (Reference) | 1.04 (0.69, 1.57) | **1.69 (1.13, 2.53)** | **2.08 (1.29, 3.34)** |  |
| 45-65 (n=10657) | 1.0 (Reference) | 1.21 (1.00, 1.47) | 1.12 (0.91, 1.39) | 1.22 (0.94, 1.59) |  |
| >65 (n=2477) | 1.0 (Reference) | 1.03 (0.73, 1.47) | 0.98 (0.66, 1.45) | 1.25 (0.78, 2.01) |  |
| **Gender** |  |  |  |  | 0.153 |
| Women (n=2963) | 1.0 (Reference) | 1.13 (0.73, 1.73) | 0.95 (0.59, 1.53) | 1.01 (0.56, 1.81) |  |
| Men (n=14826) | 1.0 (Reference) | 1.16 (0.99, 1.38) | **1.23 (1.03, 1.47)** | **1.40 (1.13, 1.75)** |  |
| **Household income per month, ¥** |  |  |  |  | 0.948 |
| <1000 (n=13137) | 1.0 (Reference) | 1.17 (0.98, 1.39) | **1.23 (1.01, 1.49)** | **1.32 (1.04, 1.68)** |  |
| ≥1000 (n=4450) | 1.0 (Reference) | 1.12 (0.80, 1.55) | 1.03 (0.72, 1.47) | 1.37 (0.90, 2.07) |  |
| **Education level** |  |  |  |  | 0.312 |
| Primary school or below (n=1670) | 1.0 (Reference) | 1.01 (0.65, 1.55) | 0.86 (0.53, 1.41) | 1.08 (0.60, 1.92) |  |
| Middle school (n=13008) | 1.0 (Reference) | **1.22 (1.02, 1.46)** | **1.27 (1.05, 1.54)** | **1.37 (1.08, 1.73)** |  |
| High or above (n=3086) | 1.0 (Reference) | 0.90 (0.58, 1.40) | 1.06 (0.65, 1.73) | 1.48 (0.81, 2.70) |  |
| **Occupation** |  |  |  |  | 0.536 |
| Coalminers (n=5273) | 1.0 (Reference) | 1.23 (0.93, 1.63) | 1.27 (0.94, 1.72) | 1.41 (0.98, 2.04) |  |
| Other blue collars (n=10966) | 1.0 (Reference) | 1.19 (0.98, 1.45) | 1.18 (0.96, 1.47) | **1.35 (1.04, 1.75)** |  |
| White collars (n=1008) | 1.0 (Reference) | 0.54 (0.28, 1.03) | 0.85 (0.43, 1.65) | 1.11 (0.49, 2.53) |  |
| **Alcohol consumption** |  |  |  |  | 0.142 |
| Never (n=10475) | 1.0 (Reference) | 1.16 (0.94, 1.42) | 1.13 (0.90, 1.41) | 1.20 (0.90, 1.58) |  |
| Moderate (n=594) | 1.0 (Reference) | 0.79 (0.39, 1.60) | 1.35 (0.65, 2.82) | 1.03 (0.38, 2.79) |  |
| Heavy (n=6177) | 1.0 (Reference) | 1.20 (0.93, 1.54) | 1.26 (0.96, 1.66) | **1.62 (1.17, 2.23)** |  |
| **Smoking status** |  |  |  |  | 0.208 |
| Never (n=11163) | 1.0 (Reference) | 1.11 (0.90, 1.36) | 1.08 (0.87, 1.35) | 1.18 (0.90, 1.54) |  |
| Former (n=835) | 1.0 (Reference) | 0.91 (0.47, 1.76) | 1.43 (0.70, 2.95) | 2.11 (0.83, 5.39) |  |
| Current (n=5777) | 1.0 (Reference) | 1.25 (0.96, 1.63) | 1.32 (1.00, 1.75) | **1.58 (1.13, 2.21)** |  |
| **Dietary quality** |  |  |  |  | 0.887 |
| Favorable (n=3230) | 1.0 (Reference) | 1.25 (0.86, 1.82) | 1.05 (0.69, 1.59) | 1.60 (0.99, 2.57) |  |
| Intermediate (n=12645) | 1.0 (Reference) | 1.13 (0.94, 1.36) | 1.14 (0.94, 1.40) | 1.26 (0.98, 1.62) |  |
| Unfavorable (n=1898) | 1.0 (Reference) | 1.21 (0.78, 1.88) | **1.59 (1.002, 2.52)** | 1.44 (0.80, 2.60) |  |
| **Physical activity** |  |  |  |  | 0.588 |
| No (n=5015) | 1.0 (Reference) | **1.35 (1.004, 1.80)** | 0.90 (0.64, 1.28) | **1.55 (1.06, 2.27)** |  |
| Occasional (n=10065) | 1.0 (Reference) | 1.06 (0.86, 1.31) | **1.28 (1.02, 1.59)** | 1.29 (0.98, 1.69) |  |
| Regular (n=2696) | 1.0 (Reference) | 1.20 (0.82, 1.75) | 1.38 (0.91, 2.08) | 1.29 (0.73, 2.27) |  |

The model was adjusted for age, gender, education level (elementary school or below; middle school; high school or above), occupation (coal miners; other blue collars; white collars), household income (<1000 ¥; ≥1000 ¥), smoking status (never; ever; current), alcohol consumption (never; moderate; heavy), physical activity (inactive; occasional; regular), dietary quality (favorable; moderate; unfavorable), antihypertensive drugs (yes or no), lipid-lowering drugs (yes or no), antidiabetic drugs (yes or no), family history of diabetes (yes or no), family history of stroke (yes or no), family history of myocardial infarction (yes or no), and mean of body mass index, high-sensitivity C-reactive protein during 2006 to 2010.

**Table S3** Hazard ratios (HRs) and 95% confidence intervals (95%CIs) of incident stroke by quartiles of metabolic parameters variability according to standard deviation.

| **Outcome** | **Variable** | **Case/Total, N** | **Follow-up duration**  **(Person-years)** | **Incidence rate, per 1000 person-years** |  | **HR (95%CI)** |  |
| --- | --- | --- | --- | --- | --- | --- | --- |
|  |  |  |  |  | **Model 1** | **Model 2** | **Model 3** |
| **Total stroke** |  | | | | | | |
|  | **0** | 260/4477 | 42366.18 | 6.137 | 1.0 (Reference) | 1.0 (Reference) | 1.0 (Reference) |
|  | **1** | 435/6798 | 63612.13 | 6.838 | 1.12 (0.96, 1.30) | 1.10 (0.95, 1.29) | 1.11 (0.94, 1.30) |
|  | **2** | 361/4539 | 41818.63 | 8.633 | **1.42 (1.21, 1.67)** | **1.41 (1.20, 1.65)** | **1.32 (1.12, 1.56)** |
|  | **≥3** | 167/1975 | 17908.67 | 9.325 | **1.54 (1.27, 1.87)** | **1.55 (1.27, 1.88)** | **1.39 (1.13, 1.71)** |
|  | ***P* for trend** | | | | **<0.001** | **<0.001** | **<0.001** |
|  | **1 point increment** | | | | **1.17 (1.11, 1.24)** | **1.18 (1.11, 1.25)** | **1.13 (1.06, 1.20)** |
| **Hemorrhagic stroke** |  | | | | | | |
|  | **0** | 37/4477 | 43173.09 | 0.857 | 1.0 (Reference) | 1.0 (Reference) | 1.0 (Reference) |
|  | **1** | 56/6798 | 64929.70 | 0.863 | 1.01 (0.67, 1.53) | 1.00 (0.66, 1.51) | 1.05 (0.69, 1.62) |
|  | **2** | 50/4539 | 42839.07 | 1.167 | 1.37 (0.89, 2.09) | 1.35 (0.89, 2.07) | 1.11 (0.70, 1.77) |
|  | **≥3** | 25/1975 | 18371.78 | 1.361 | 1.60 (0.96, 2.65) | 1.60 (0.96, 2.66) | 1.50 (0.88, 2.57) |
|  | ***P* for trend** | | | | **0.024** | **0.023** | 0.154 |
|  | **1 point increment** | | | | **1.19 (1.02, 1.40)** | **1.19 (1.02, 1.40)** | 1.12 (0.95, 1.33) |
| **Ischemic stroke** |  | | | | | | |
|  | **0** | 233/4477 | 42442.85 | 5.490 | 1.0 (Reference) | 1.0 (Reference) | 1.0 (Reference) |
|  | **1** | 393/6798 | 63746.35 | 6.165 | 1.13 (0.96, 1.32) | 1.11 (0.95, 1.31) | 1.11 (0.94, 1.31) |
|  | **2** | 321/4539 | 41937.31 | 7.654 | **1.41 (1.19, 1.67)** | **1.40 (1.18, 1.65)** | **1.36 (1.12, 1.59)** |
|  | **≥3** | 148/1975 | 17962.19 | 8.240 | **1.52 (1.24, 1.87)** | **1.53 (1.25, 1.88)** | **1.37 (1.10, 1.70)** |
|  | ***P* for trend** | | | | **<0.001** | **<0.001** | **<0.001** |
|  | **1 point increment** | | | | **1.17 (1.10, 1.24)** | **1.17 (1.10, 1.24)** | **1.13 (1.06, 1.20)** |

Model 1 was non-adjusted;

Model 2 was adjusted for age and gender;

Model 3 was further adjusted for education level (elementary school or below; middle school; high school or above), occupation (coal miners; other blue collars; white collars), household income (<1000 ¥; ≥1000 ¥), smoking status (never; ever; current), alcohol consumption (never; moderate; heavy), physical activity (inactive; occasional; regular), dietary quality (favorable; moderate; unfavorable), antihypertensive drugs (yes or no), lipid-lowering drugs (yes or no), antidiabetic drugs (yes or no), family history of diabetes (yes or no), family history of stroke (yes or no), family history of myocardial infarction (yes or no), and mean of body mass index, high-sensitivity C-reactive protein during 2006 to 2010.

**Table S4** Hazard ratios (HRs) and 95% confidence intervals (95%CIs) of incident stroke by quartiles of metabolic parameters variability according to average real variability.

| **Outcome** | **Variable** | **Case/Total, N** | **Follow-up duration**  **(Person-years)** | **Incidence rate, per 1000 person-years** |  | **HR (95%CI)** |  |
| --- | --- | --- | --- | --- | --- | --- | --- |
|  |  |  |  |  | **Model 1** | **Model 2** | **Model 3** |
| **Total stroke** |  | | | | | | |
|  | **0** | 284/4774 | 45242.63 | 6.277 | 1.0 (Reference) | 1.0 (Reference) | 1.0 (Reference) |
|  | **1** | 444/6784 | 63358.67 | 7.008 | 1.12 (0.97, 1.30) | 1.10 (0.95, 1.28) | 1.07 (0.92, 1.25) |
|  | **2** | 344/4400 | 40625.89 | 8.468 | **1.36 (1.16, 1.59)** | **1.35 (1.15, 1.58)** | **1.25 (1.06, 1.47)** |
|  | **≥3** | 151/1831 | 16478.41 | 9.164 | **1.48 (1.22, 1.80)** | **1.47 (1.20, 1.79)** | **1.30 (1.06, 1.61)** |
|  | ***P* for trend** | | | | **<0.001** | **<0.001** | **0.001** |
|  | **1 point increment** | | | | **1.15 (1.09, 1.22)** | **1.15 (1.09, 1.22)** | **1.11 (1.04, 1.18)** |
| **Hemorrhagic stroke** |  | | | | | | |
|  | **0** | 43/4774 | 46132.51 | 0.932 | 1.0 (Reference) | 1.0 (Reference) | 1.0 (Reference) |
|  | **1** | 57/6784 | 64626.43 | 0.882 | 0.95 (0.64, 1.41) | 0.94 (0.63, 1.39) | 0.86 (0.57, 1.30) |
|  | **2** | 43/4400 | 41668.32 | 1.032 | 1.11 (0.73, 1.70) | 1.10 (0.72, 1.68) | 0.95 (0.61, 1.48) |
|  | **≥3** | 25/1831 | 16886.38 | 1.481 | 1.60 (0.98, 2.62) | 1.58 (0.97, 2.59) | 1.37 (0.81, 2.33) |
|  | ***P* for trend** | | | | 0.063 | 0.069 | 0.278 |
|  | **1 point increment** | | | | 1.15 (0.98, 1.35) | 1.15 (0.98, 1.34) | 1.08 (0.91, 1.28) |
| **Ischemic stroke** |  | | | | | | |
|  | **0** | 254/4774 | 45330.48 | 5.603 | 1.0 (Reference) | 1.0 (Reference) | 1.0 (Reference) |
|  | **1** | 399/6784 | 63516.01 | 6.282 | 1.13 (0.96, 1.32) | 1.11 (0.95, 1.30) | 1.08 (0.92, 1.27) |
|  | **2** | 309/4400 | 40711.54 | 7.590 | **1.36 (1.16, 1.61)** | **1.35 (1.15, 1.60)** | **1.27 (1.07, 1.51)** |
|  | **≥3** | 133/1831 | 16530.66 | 8.046 | **1.46 (1.18, 1.80)** | **1.44 (1.17, 1.78)** | **1.30 (1.04, 1.62)** |
|  | ***P* for trend** | | | | **<0.001** | **<0.001** | **0.002** |
|  | **1 point increment** | | | | **1.15 (1.08, 1.22)** | **1.15 (1.08, 1.22)** | **1.11 (1.04, 1.18)** |

Model 1 was non-adjusted;

Model 2 was adjusted for age and gender;

Model 3 was further adjusted for education level (elementary school or below; middle school; high school or above), occupation (coal miners; other blue collars; white collars), household income (<1000 ¥; ≥1000 ¥), smoking status (never; ever; current), alcohol consumption (never; moderate; heavy), physical activity (inactive; occasional; regular), dietary quality (favorable; moderate; unfavorable), antihypertensive drugs (yes or no), lipid-lowering drugs (yes or no), antidiabetic drugs (yes or no), family history of diabetes (yes or no), family history of stroke (yes or no), family history of myocardial infarction (yes or no), and mean of body mass index, high-sensitivity C-reactive protein during 2006 to 2010.

**Table S5** Hazard ratios (HRs) and 95% confidence intervals (95%CIs) of incident stroke by quartiles of metabolic parameters variability according to variability independent of the mean.

| **Outcome** | **Variable** | **Case/Total, N** | **Follow-up duration**  **(Person-years)** | **Incidence rate, per 1000 person-years** |  | **HR (95%CI)** |  |
| --- | --- | --- | --- | --- | --- | --- | --- |
|  |  |  |  |  | **Model 1** | **Model 2** | **Model 3** |
| **Total stroke** |  | | | | | | |
|  | **0** | 293/4354 | 40984.24 | 7.149 | 1.0 (Reference) | 1.0 (Reference) | 1.0 (Reference) |
|  | **1** | 465/6934 | 64800.44 | 7.176 | 1.01 (0.87, 1.16) | 1.00 (0.86, 1.16) | 1.03 (0.89, 1.20) |
|  | **2** | 324/4578 | 42370.73 | 7.647 | 1.07 (0.92, 1.26) | 1.07 (0.91, 1.25) | 1.08 (0.91, 1.27) |
|  | **≥3** | 141/1923 | 17550.20 | 8.034 | 1.13 (0.93, 1.39) | 1.13 (0.92, 1.38) | 1.17 (0.95, 1.44) |
|  | ***P* for trend** | | | | 0.141 | 0.160 | 0.110 |
|  | **1 point increment** | | | | 1.04 (0.98, 1.11) | 1.04 (0.98, 1.11) | 1.05 (0.99, 1.12) |
| **Hemorrhagic stroke** |  | | | | | | |
|  | **0** | 39/4354 | 41816.87 | 0.932 | 1.0 (Reference) | 1.0 (Reference) | 1.0 (Reference) |
|  | **1** | 56/6934 | 66186.66 | 0.846 | 0.91 (0.60, 1.37) | 0.91 (0.60, 1.36) | 0.95 (0.62, 1.46) |
|  | **2** | 52/4578 | 43341.65 | 1.120 | 1.29 (0.85, 1.96) | 1.29 (0.85, 1.95) | 1.24 (0.79, 1.93) |
|  | **≥3** | 21/1923 | 17938.17 | 1.171 | 1.26 (0.74, 2.15) | 1.26 (0.74, 2.14) | 1.21 (0.68, 2.14) |
|  | ***P* for trend** | | | | 0.120 | 0.123 | 0.245 |
|  | **1 point increment** | | | | 1.13 (0.96, 1.32) | 1.13 (0.96, 1.32) | 1.10 (0.93, 1.30) |
| **Ischemic stroke** |  | | | | | | |
|  | **0** | 266/4354 | 41023.93 | 6.479 | 1.0 (Reference) | 1.0 (Reference) | 1.0 (Reference) |
|  | **1** | 420/6934 | 64936.69 | 6.468 | 1.00 (0.86, 1.17) | 0.99 (0.85, 1.16) | 1.02 (0.87, 1.20) |
|  | **2** | 283/4578 | 42499.27 | 6.659 | 1.03 (0.87, 1.22) | 1.03 (0.87, 1.21) | 1.04 (0.88, 1.24) |
|  | **≥3** | 126/1923 | 17598.51 | 7.160 | 1.11 (0.90, 1.38) | 1.11 (0.90, 1.37) | 1.17 (0.94, 1.45) |
|  | ***P* for trend** | | | | 0.334 | 0.370 | 0.222 |
|  | **1 point increment** | | | | 1.03 (0.97, 1.09) | 1.03 (0.97, 1.09) | 1.04 (0.98, 1.11) |

Model 1 was non-adjusted;

Model 2 was adjusted for age and gender;

Model 3 was further adjusted for education level (elementary school or below; middle school; high school or above), occupation (coal miners; other blue collars; white collars), household income (<1000 ¥; ≥1000 ¥), smoking status (never; ever; current), alcohol consumption (never; moderate; heavy), physical activity (inactive; occasional; regular), dietary quality (favorable; moderate; unfavorable), antihypertensive drugs (yes or no), lipid-lowering drugs (yes or no), antidiabetic drugs (yes or no), family history of diabetes (yes or no), family history of stroke (yes or no), family history of myocardial infarction (yes or no), and mean of body mass index, high-sensitivity C-reactive protein during 2006 to 2010.

**Table S6** Sensitivity analysis of excluding participants with the use of anti-hypertensive drugs (n=3681).

| **Outcome** | **Variable** | **Case/Total, N** | **Follow-up duration**  **(Person-years)** | **Incidence rate, per 1000 person-years** |  | **HR (95%CI)** |  |
| --- | --- | --- | --- | --- | --- | --- | --- |
|  |  |  |  |  | **Model1** | **Model2** | **Model3** |
| **Total stroke** |  | | | | | | |
|  | **0** | 205/3505 | 33228.34 | 6.169 | 1.0 (Reference) | 1.0 (Reference) | 1.0 (Reference) |
|  | **1** | 341/5456 | 51256.28 | 6.653 | 1.08 (0.91, 1.29) | 1.08 (0.91, 1.29) | 1.08 (0.91, 1.30) |
|  | **2** | 230/3573 | 33386.32 | 6.889 | 1.12 (0.93, 1.36) | 1.13 (0.94, 1.37) | 1.10 (0.91, 1.34) |
|  | **≥3** | 121/1574 | 14370.62 | 8.420 | **1.38 (1.10, 1.73)** | **1.37 (1.09, 1.72)** | **1.31 (1.04, 1.66)** |
|  | ***P* for trend** | | | | **0.007** | **0.007** | **0.031** |
|  | **1 point increment** | | | | **1.10 (1.02, 1.17)** | **1.10 (1.02, 1.17)** | **1.08 (1.003, 1.16)** |
| **Hemorrhagic stroke** |  | | | | | | |
|  | **0** | 30/3505 | 33837.54 | 0.887 | 1.0 (Reference) | 1.0 (Reference) | 1.0 (Reference) |
|  | **1** | 44/5456 | 52284.25 | 0.842 | 0.95 (0.60, 1.51) | 0.95 (0.60, 1.51) | 0.93 (0.57, 1.50) |
|  | **2** | 39/3573 | 34028.92 | 1.146 | 1.30 (0.81, 2.09) | 1.30 (0.81, 2.09) | 1.22 (0.74, 2.01) |
|  | **≥3** | 20/1574 | 14696.51 | 1.361 | 1.55 (0.88, 2.72) | 1.53 (0.87, 2.69) | 1.54 (0.86, 2.77) |
|  | ***P* for trend** | | | | 0.056 | 0.061 | 0.077 |
|  | **1 point increment** | | | | 1.18 (0.99, 1.41) | 1.18 (0.99, 1.40) | 1.17 (0.97, 1.41) |
| **Ischemic stroke** |  | | | | | | |
|  | **0** | 183/3505 | 33287.25 | 5.498 | 1.0 (Reference) | 1.0 (Reference) | 1.0 (Reference) |
|  | **1** | 306/5456 | 51356.43 | 5.958 | 1.09 (0.90, 1.31) | 1.09 (0.90, 1.30) | 1.10 (0.91, 1.33) |
|  | **2** | 200/3573 | 33483.15 | 5.973 | 1.09 (0.89, 1.33) | 1.10 (0.90, 1.35) | 1.09 (0.88, 1.34) |
|  | **≥3** | 107/1574 | 14417.19 | 7.422 | **1.36 (1.07, 1.73)** | **1.36 (1.07, 1.72)** | **1.29 (1.01, 1.66)** |
|  | ***P* for trend** | | | | **0.022** | **0.021** | **0.065** |
|  | **1 point increment** | | | | **1.09 (1.01, 1.17)** | **1.09 (1.01, 1.17)** | 1.07 (0.99, 1.15) |

Model 1 was non-adjusted;

Model 2 was adjusted for age and gender;

Model 3 was further adjusted for education level (elementary school or below; middle school; high school or above), occupation (coal miners; other blue collars; white collars), household income (<1000 ¥; ≥1000 ¥), smoking status (never; ever; current), alcohol consumption (never; moderate; heavy), physical activity (inactive; occasional; regular), dietary quality (favorable; moderate; unfavorable), lipid-lowering drugs (yes or no), antidiabetic drugs (yes or no), family history of diabetes (yes or no), family history of stroke (yes or no), family history of myocardial infarction (yes or no), and mean of body mass index, high-sensitivity C-reactive protein during 2006 to 2010.

**Table S7** Sensitivity analysis of excluding participants with the use of antidiabetic drugs (n=791).

| **Outcome** | **Variable** | **Case/Total, N** | **Follow-up duration**  **(Person-years)** | **Incidence rate, per 1000 person-years** |  | **HR (95%CI)** |  |
| --- | --- | --- | --- | --- | --- | --- | --- |
|  |  |  |  |  | **Model1** | **Model2** | **Model3** |
| **Total stroke** |  | | | | | | |
|  | **0** | 251/4191 | 39632.89 | 6.333 | 1.0 (Reference) | 1.0 (Reference) | 1.0 (Reference) |
|  | **1** | 445/6599 | 61624.70 | 7.221 | 1.14 (0.98, 1.34) | 1.14 (0.98, 1.33) | 1.15 (0.98, 1.35) |
|  | **2** | 289/4343 | 40521.90 | 7.132 | 1.13 (0.96, 1.34) | 1.14 (0.96, 1.35) | 1.11 (0.93, 1.32) |
|  | **≥3** | 148/1865 | 17076.35 | 8.667 | **1.38 (1.13, 1.69)** | **1.39 (1.14, 1.71)** | **1.38 (1.12, 1.70)** |
|  | ***P* for trend** | | | | **0.005** | **0.004** | **0.010** |
|  | **1 point increment** | | | | **1.09 (1.02, 1.16)** | **1.09 (1.03, 1.16)** | **1.08 (1.02, 1.15)** |
| **Hemorrhagic stroke** |  | | | | | | |
|  | **0** | 33/4191 | 40391.02 | 0.817 | 1.0 (Reference) | 1.0 (Reference) | 1.0 (Reference) |
|  | **1** | 55/6599 | 62965.73 | 0.874 | 1.07 (0.70, 1.65) | 1.07 (0.69, 1.64) | 1.06 (0.68, 1.66) |
|  | **2** | 45/4343 | 41348.89 | 1.088 | 1.34 (0.85, 2.10) | 1.34 (0.86, 2.10) | 1.16 (0.72, 1.86) |
|  | **≥3** | 23/1865 | 17483.54 | 1.316 | 1.62 (0.95, 2.76) | 1.63 (0.96, 2.77) | 1.59 (0.92, 2.76) |
|  | ***P* for trend** | | | | **0.040** | **0.037** | 0.095 |
|  | **1 point increment** | | | | **1.18 (1.005, 1.39)** | **1.19 (1.01, 1.40)** | 1.15 (0.97, 1.36) |
| **Ischemic stroke** |  | | | | | | |
|  | **0** | 228/4191 | 39697.02 | 5.744 | 1.0 (Reference) | 1.0 (Reference) | 1.0 (Reference) |
|  | **1** | 400/6599 | 61760.28 | 6.477 | 1.13 (0.96, 1.33) | 1.13 (0.96, 1.33) | 1.14 (0.97, 1.35) |
|  | **2** | 255/4343 | 40627.41 | 6.277 | 1.10 (0.92, 1.31) | 1.11 (0.92, 1.32) | 1.10 (0.92, 1.33) |
|  | **≥3** | 131/1865 | 17120.81 | 7.652 | **1.35 (1.09, 1.67)** | **1.36 (1.09, 1.68)** | **1.35 (1.08, 1.69)** |
|  | ***P* for trend** | | | | **0.021** | **0.016** | **0.023** |
|  | **1 point increment** | | | | **1.08 (1.01, 1.15)** | **1.08 (1.01, 1.15)** | **1.08 (1.01, 1.15)** |

Model 1 was non-adjusted;

Model 2 was adjusted for age and gender;

Model 3 was further adjusted for education level (elementary school or below; middle school; high school or above), occupation (coal miners; other blue collars; white collars), household income (<1000 ¥; ≥1000 ¥), smoking status (never; ever; current), alcohol consumption (never; moderate; heavy), physical activity (inactive; occasional; regular), dietary quality (favorable; moderate; unfavorable), anti-hypertensive drugs (yes or no), lipid-lowering drugs (yes or no), family history of diabetes (yes or no), family history of stroke (yes or no), family history of myocardial infarction (yes or no), and mean of body mass index, high-sensitivity C-reactive protein during 2006 to 2010.

**Table S8** Sensitivity analysis of excluding participants with the use of lipid-lowering drugs (n=270).

| **Outcome** | **Variable** | **Case/Total, N** | **Follow-up duration**  **(Person-years)** | **Incidence rate, per 1000 person-years** |  | **HR (95%CI)** |  |
| --- | --- | --- | --- | --- | --- | --- | --- |
|  |  |  |  |  | **Model1** | **Model2** | **Model3** |
| **Total stroke** |  | | | | | | |
|  | **0** | 263/4339 | 40930.37 | 6.426 | 1.0 (Reference) | 1.0 (Reference) | 1.0 (Reference) |
|  | **1** | 464/6766 | 63049.81 | 7.359 | 1.15 (0.99, 1.34) | 1.15 (0.99, 1.33) | 1.14 (0.98, 1.34) |
|  | **2** | 322/4479 | 41564.99 | 7.747 | **1.21 (1.03, 1.43)** | **1.22 (1.03, 1.43)** | 1.17 (0.98, 1.38) |
|  | **≥3** | 158/1935 | 17634.61 | 8.960 | **1.41 (1.16, 1.72)** | **1.41 (1.16, 1.72)** | **1.33 (1.08, 1.64)** |
|  | ***P* for trend** | | | | **0.001** | **<0.001** | **0.007** |
|  | **1 point increment** | | | | **1.11 (1.05, 1.18)** | **1.11 (1.05, 1.18)** | **1.09 (1.02, 1.16)** |
| **Hemorrhagic stroke** |  | | | | | | |
|  | **0** | 36/4339 | 41728.26 | 0.863 | 1.0 (Reference) | 1.0 (Reference) | 1.0 (Reference) |
|  | **1** | 58/6766 | 64434.68 | 0.900 | 1.05 (0.69, 1.58) | 1.04 (0.69, 1.58) | 1.05 (0.68, 1.61) |
|  | **2** | 49/4479 | 42490.29 | 1.153 | 1.34 (0.87, 2.06) | 1.34 (0.87, 2.07) | 1.20 (0.75, 1.89) |
|  | **≥3** | 25/1935 | 18071.04 | 1.383 | 1.61 (0.97, 2.69) | 1.61 (0.97, 2.69) | 1.55 (0.91, 2.65) |
|  | ***P* for trend** | | | | **0.029** | **0.028** | 0.085 |
|  | **1 point increment** | | | | **1.19 (1.01, 1.39)** | **1.19 (1.01, 1.39)** | 1.15 (0.97, 1.36) |
| **Ischemic stroke** |  | | | | | | |
|  | **0** | 237/4339 | 41006.03 | 5.780 | 1.0 (Reference) | 1.0 (Reference) | 1.0 (Reference) |
|  | **1** | 418/6766 | 63185.97 | 6.615 | 1.15 (0.98, 1.35) | 1.15 (0.98, 1.34) | 1.14 (0.97, 1.35) |
|  | **2** | 285/4479 | 41682.71 | 6.837 | **1.19 (1.002, 1.41)** | **1.19 (1.004, 1.42)** | 1.16 (0.97, 1.39) |
|  | **≥3** | 139/1935 | 17688.17 | 7.858 | **1.37 (1.11, 1.69)** | **1.38 (1.12, 1.70)** | **1.30 (1.04, 1.62)** |
|  | ***P* for trend** | | | | **0.003** | **0.003** | **0.019** |
|  | **1 point increment** | | | | **1.10 (1.03, 1.17)** | **1.10 (1.03, 1.17)** | **1.08 (1.01, 1.15)** |

Model 1 was non-adjusted;

Model 2 was adjusted for age and gender;

Model 3 was further adjusted for education level (elementary school or below; middle school; high school or above), occupation (coal miners; other blue collars; white collars), household income (<1000 ¥; ≥1000 ¥), smoking status (never; ever; current), alcohol consumption (never; moderate; heavy), physical activity (inactive; occasional; regular), dietary quality (favorable; moderate; unfavorable), anti-hypertensive drugs (yes or no), antidiabetic drugs (yes or no), family history of diabetes (yes or no), family history of stroke (yes or no), family history of myocardial infarction (yes or no), and mean of body mass index, high-sensitivity C-reactive protein during 2006 to 2010.

**Table S9** Sensitivity analysis of excluding participants who had the incidence outcome within the first 2 years.

| **Outcome** | **Variable** | **Case/Total, N** | **Follow-up duration**  **(Person-years)** | **Incidence rate, per 1000 person-years** |  | **HR (95%CI)** |  |
| --- | --- | --- | --- | --- | --- | --- | --- |
|  |  |  |  |  | **Model1** | **Model2** | **Model3** |
| **Total stroke** |  | | | | | | |
|  | **0** | 262/4400 | 41538.88 | 6.307 | 1.0 (Reference) | 1.0 (Reference) | 1.0 (Reference) |
|  | **1** | 464/6861 | 64024.32 | 7.234 | 1.15 (0.99, 1.34) | 1.15 (0.99, 1.34) | 1.15 (0.98, 1.34) |
|  | **2** | 326/4558 | 42294.10 | 7.732 | **1.23 (1.05, 1.45)** | **1.23 (1.05, 1.45)** | **1.19 (1.003, 1.41)** |
|  | **≥3** | 159/1958 | 17828.14 | 8.910 | **1.43 (1.17, 1.74)** | **1.44 (1.18, 1.75)** | **1.35 (1.11, 1.67)** |
|  | ***P* for trend** | | | | **<0.001** | **<0.001** | **0.003** |
|  | **1 point increment** | | | | **1.12 (1.05, 1.18)** | **1.12 (1.05, 1.19)** | **1.09 (1.03, 1.16)** |
| **Hemorrhagic stroke** |  | | | | | | |
|  | **0** | 36/4400 | 42323.14 | 0.851 | 1.0 (Reference) | 1.0 (Reference) | 1.0 (Reference) |
|  | **1** | 58/6861 | 65394.40 | 0.887 | 1.05 (0.69, 1.58) | 1.04 (0.69, 1.58) | 1.05 (0.68, 1.62) |
|  | **2** | 47/4558 | 43244.66 | 1.087 | 1.28 (0.83, 1.98) | 1.28 (0.83, 1.98) | 1.14 (0.72, 1.81) |
|  | **≥3** | 25/1958 | 18269.81 | 1.368 | 1.62 (0.97, 2.70) | 1.62 (0.97, 2.70) | 1.56 (0.91, 2.66) |
|  | ***P* for trend** | | | | **0.039** | **0.037** | 0.109 |
|  | **1 point increment** | | | | **1.18 (1.005, 1.38)** | **1.18 (1.006, 1.38)** | 1.14 (0.96, 1.35) |
| **Ischemic stroke** |  | | | | | | |
|  | **0** | 236/4400 | 41614.54 | 5.671 | 1.0 (Reference) | 1.0 (Reference) | 1.0 (Reference) |
|  | **1** | 418/6861 | 64164.50 | 6.515 | 1.15 (0.98, 1.35) | 1.15 (0.98, 1.35) | 1.15 (0.98, 1.35) |
|  | **2** | 291/4558 | 42407.07 | 6.862 | **1.22 (1.03, 1.45)** | **1.22 (1.03, 1.45)** | 1.19 (1.00, 1.43) |
|  | **≥3** | 140/1958 | 17881.70 | 7.829 | **1.40 (1.13, 1.72)** | **1.40 (1.14, 1.73)** | **1.33 (1.06, 1.65)** |
|  | ***P* for trend** | | | | **0.001** | **0.001** | **0.008** |
|  | **1 point increment** | | | | **1.11 (1.04, 1.18)** | **1.11 (1.04, 1.18)** | **1.09 (1.02, 1.16)** |

Model 1 was non-adjusted;

Model 2 was adjusted for age and gender;

Model 3 was further adjusted for education level (elementary school or below; middle school; high school or above), occupation (coal miners; other blue collars; white collars), household income (<1000 ¥; ≥1000 ¥), smoking status (never; ever; current), alcohol consumption (never; moderate; heavy), physical activity (inactive; occasional; regular), dietary quality (favorable; moderate; unfavorable), antihypertensive drugs (yes or no), lipid-lowering drugs (yes or no), antidiabetic drugs (yes or no), family history of diabetes (yes or no), family history of stroke (yes or no), family history of myocardial infarction (yes or no), and mean of body mass index, high-sensitivity C-reactive protein during 2006 to 2010.

**Table S10** Sensitivity analysis of excluding participants who developed atrial fibrillation.

| **Outcome** | **Variable** | **Case/Total, N** | **Follow-up duration**  **(Person-years)** | **Incidence rate, per 1000 person-years** |  | **HR (95%CI)** |  |
| --- | --- | --- | --- | --- | --- | --- | --- |
|  |  |  |  |  | **Model1** | **Model2** | **Model3** |
| **Total stroke** |  | | | | | | |
|  | **0** | 250/4329 | 40917.70 | 6.110 | 1.0 (Reference) | 1.0 (Reference) | 1.0 (Reference) |
|  | **1** | 461/6735 | 62894.05 | 7.330 | **1.20 (1.03, 1.40)** | **1.20 (1.03, 1.40)** | **1.20 (1.02, 1.41)** |
|  | **2** | 317/4452 | 41366.46 | 7.663 | **1.26 (1.07, 1.49)** | **1.26 (1.07, 1.49)** | **1.22 (1.02, 1.44)** |
|  | **≥3** | 154/1912 | 17461.49 | 8.819 | **1.46 (1.19, 1.78)** | **1.47 (1.20, 1.80)** | **1.38 (1.12, 1.71)** |
|  | ***P* for trend** | | | | **<0.001** | **<0.001** | **0.003** |
|  | **1 point increment** | | | | **1.12 (1.06, 1.19)** | **1.12 (1.06, 1.19)** | **1.10 (1.03, 1.17)** |
| **Hemorrhagic stroke** |  | | | | | | |
|  | **0** | 36/4329 | 41671.96 | 0.864 | 1.0 (Reference) | 1.0 (Reference) | 1.0 (Reference) |
|  | **1** | 58/6735 | 64277.26 | 0.902 | 1.05 (0.69, 1.59) | 1.04 (0.69, 1.58) | 1.05 (0.68, 1.62) |
|  | **2** | 48/4452 | 42280.04 | 1.135 | 1.32 (0.86, 2.03) | 1.32 (0.86, 2.03) | 1.18 (0.75, 1.88) |
|  | **≥3** | 25/1912 | 17888.82 | 1.340 | 1.63 (0.98, 2.71) | 1.64 (0.98, 2.72) | 1.58 (0.93, 2.71) |
|  | ***P* for trend** | | | | **0.030** | **0.028** | 0.078 |
|  | **1 point increment** | | | | **1.18 (1.01, 1.39)** | **1.19 (1.01, 1.39)** | 1.15 (0.97, 1.36) |
| **Ischemic stroke** |  | | | | | | |
|  | **0** | 224/4329 | 40993.36 | 5.464 | 1.0 (Reference) | 1.0 (Reference) | 1.0 (Reference) |
|  | **1** | 414/6735 | 63034.27 | 6.568 | **1.21 (1.03, 1.42)** | **1.20 (1.02, 1.41)** | **1.20 (1.01, 1.42)** |
|  | **2** | 281/4452 | 41480.13 | 6.774 | **1.25 (1.05, 1.49)** | **1.25 (1.05, 1.50)** | **1.22 (1.02, 1.46)** |
|  | **≥3** | 135/1912 | 17515.05 | 7.708 | **1.43 (1.15, 1.76)** | **1.44 (1.16, 1.78)** | **1.35 (1.08, 1.69)** |
|  | ***P* for trend** | | | | **0.001** | **0.001** | **0.008** |
|  | **1 point increment** | | | | **1.11 (1.04, 1.18)** | **1.11 (1.05, 1.19)** | **1.09 (1.02, 1.17)** |

Model 1 was non-adjusted;

Model 2 was adjusted for age and gender;

Model 3 was further adjusted for education level (elementary school or below; middle school; high school or above), occupation (coal miners; other blue collars; white collars), household income (<1000 ¥; ≥1000 ¥), smoking status (never; ever; current), alcohol consumption (never; moderate; heavy), physical activity (inactive; occasional; regular), dietary quality (favorable; moderate; unfavorable), antihypertensive drugs (yes or no), lipid-lowering drugs (yes or no), antidiabetic drugs (yes or no), family history of diabetes (yes or no), family history of stroke (yes or no), family history of myocardial infarction (yes or no), and mean of body mass index, high-sensitivity C-reactive protein during 2006 to 2010.

**Table S11** Sensitivity analysis of excluding participant who developed cancer during 2006 to 2010.

| **Outcome** | **Variable** | **Case/Total, N** | **Follow-up duration**  **(Person-years)** | **Incidence rate, per 1000 person-years** |  | **HR (95%CI)** |  |
| --- | --- | --- | --- | --- | --- | --- | --- |
|  |  |  |  |  | **Model1** | **Model2** | **Model3** |
| **Total stroke** |  | | | | | | |
|  | **0** | 264/4347 | 41128.41 | 6.419 | 1.0 (Reference) | 1.0 (Reference) | 1.0 (Reference) |
|  | **1** | 466/6744 | 63257.32 | 7.367 | 1.15 (0.99, 1.34) | 1.15 (0.99, 1.33) | 1.14 (0.98, 1.34) |
|  | **2** | 325/4469 | 41723.98 | 7.789 | **1.22 (1.04, 1.44)** | **1.22 (1.04, 1.44)** | 1.18 (1.00, 1.40) |
|  | **≥3** | 156/1916 | 17622.99 | 8.852 | **1.39 (1.14, 1.70)** | **1.40 (1.15, 1.70)** | **1.33 (1.08, 1.63)** |
|  | ***P* for trend** | | | | **0.001** | **0.001** | **0.006** |
|  | **1 point increment** | | | | **1.11 (1.04, 1.17)** | **1.11 (1.05, 1.18)** | **1.09 (1.02, 1.16)** |
| **Hemorrhagic stroke** |  | | | | | | |
|  | **0** | 35/4347 | 41931.59 | 0.835 | 1.0 (Reference) | 1.0 (Reference) | 1.0 (Reference) |
|  | **1** | 58/6744 | 64652.48 | 0.897 | 1.08 (0.71, 1.64) | 1.07 (0.70, 1.63) | 1.08 (0.70, 1.68) |
|  | **2** | 47/4469 | 42675.06 | 1.101 | 1.32 (0.86, 2.05) | 1.33 (0.86, 2.05) | 1.18 (0.74, 1.89) |
|  | **≥3** | 24/1916 | 18059.29 | 1.329 | 1.60 (0.95, 2.69) | 1.61 (0.95, 2.70) | 1.54 (0.89, 2.66) |
|  | ***P* for trend** | | | | **0.042** | **0.040** | 0.111 |
|  | **1 point increment** | | | | **1.18 (1.003, 1.38)** | **1.18 (1.004, 1.38)** | 1.14 (0.96, 1.35) |
| **Ischemic stroke** |  | | | | | | |
|  | **0** | 238/4347 | 41204.07 | 5.776 | 1.0 (Reference) | 1.0 (Reference) | 1.0 (Reference) |
|  | **1** | 419/6744 | 63397.53 | 6.609 | 1.15 (0.98, 1.35) | 1.14 (0.97, 1.34) | 1.14 (0.97, 1.34) |
|  | **2** | 290/4469 | 41837.64 | 6.932 | **1.21 (1.02, 1.43)** | **1.21 (1.02, 1.44)** | 1.18 (0.99, 1.41) |
|  | **≥3** | 138/1916 | 17675.26 | 7.808 | **1.36 (1.11, 1.68)** | **1.37 (1.11, 1.69)** | **1.30 (1.05, 1.62)** |
|  | ***P* for trend** | | | | **0.003** | **0.002** | **0.012** |
|  | **1 point increment** | | | | **1.10 (1.03, 1.17)** | **1.10 (1.04, 1.17)** | **1.08 (1.02, 1.16)** |

Model 1 was non-adjusted;

Model 2 was adjusted for age and gender;

Model 3 was further adjusted for education level (elementary school or below; middle school; high school or above), occupation (coal miners; other blue collars; white collars), household income (<1000 ¥; ≥1000 ¥), smoking status (never; ever; current), alcohol consumption (never; moderate; heavy), physical activity (inactive; occasional; regular), dietary quality (favorable; moderate; unfavorable), antihypertensive drugs (yes or no), lipid-lowering drugs (yes or no), antidiabetic drugs (yes or no), family history of diabetes (yes or no), family history of stroke (yes or no), family history of myocardial infarction (yes or no), and mean of body mass index, high-sensitivity C-reactive protein during 2006 to 2010.

**Table S12** Sensitivity analysis of DBP by SBP formed the metabolic syndrome parameter variability.

| **Outcome** | **Variable** | **Case/Total, N** | **Follow-up duration**  **(Person-years)** | **Incidence rate, per 1000 person-years** |  | **HR (95%CI)** |  |
| --- | --- | --- | --- | --- | --- | --- | --- |
|  |  |  |  |  | **Model1** | **Model2** | **Model3** |
| **Total stroke** |  | | | | | | |
|  | **0** | 267/4375 | 41168.75 | 6.486 | 1.0 (Reference) | 1.0 (Reference) | 1.0 (Reference) |
|  | **1** | 483/6963 | 65005.25 | 7.430 | 1.15 (0.99, 1.33) | 1.14 (0.99, 1.33) | 1.14 (0.98, 1.33) |
|  | **2** | 328/4564 | 42260.72 | 7.761 | **1.20 (1.02, 1.42)** | **1.20 (1.02, 1.41)** | 1.13 (0.96, 1.34) |
|  | **≥3** | 145/1887 | 17270.89 | 8.396 | **1.31 (1.07, 1.60)** | **1.31 (1.07, 1.60)** | 1.22 (0.99, 1.51) |
|  | ***P* for trend** | | | | **0.006** | **0.006** | 0.066 |
|  | **1 point increment** | | | | **1.09 (1.03, 1.15)** | **1.09 (1.03, 1.16)** | 1.06 (0.995, 1.13) |
| **Hemorrhagic stroke** |  | | | | | | |
|  | **0** | 42/4375 | 41954.49 | 0.100 | 1.0 (Reference) | 1.0 (Reference) | 1.0 (Reference) |
|  | **1** | 58/6963 | 66490.74 | 0.087 | 0.87 (0.59, 1.30) | 0.87 (0.58, 1.29) | 0.83 (0.55, 1.25) |
|  | **2** | 49/4564 | 43175.44 | 0.113 | 1.14 (0.75, 1.72) | 1.14 (0.75, 1.72) | 0.97 (0.63, 1.51) |
|  | **≥3** | 19/1887 | 17692.96 | 0.107 | 1.08 (0.63, 1.85) | 1.08 (0.63, 1.86) | 0.96 (0.54, 1.71) |
|  | ***P* for trend** | | | | 0.437 | 0.431 | 0.887 |
|  | **1 point increment** | | | | 1.06 (0.90, 1.25) | 1.06 (0.91, 1.25) | 1.00 (0.85, 1.19) |
| **Ischemic stroke** |  | | | | | | |
|  | **0** | 234/4375 | 41282.70 | 5.668 | 1.0 (Reference) | 1.0 (Reference) | 1.0 (Reference) |
|  | **1** | 440/6963 | 65133.19 | 6.755 | **1.19 (1.02, 1.40)** | **1.19 (1.02, 1.40)** | **1.20 (1.02, 1.41)** |
|  | **2** | 292/4564 | 42359.12 | 6.893 | **1.22 (1.03, 1.45)** | **1.22 (1.03, 1.45)** | 1.18 (0.98, 1.41) |
|  | **≥3** | 129/1887 | 17313.69 | 7.451 | **1.33 (1.07, 1.64)** | **1.33 (1.07, 1.65)** | **1.26 (1.003, 1.57)** |
|  | ***P* for trend** | | | | **0.009** | **0.008** | 0.053 |
|  | **1 point increment** | | | | **1.09 (1.02, 1.16)** | **1.09 (1.03, 1.16)** | 1.07 (0.9998, 1.14) |

Model 1 was non-adjusted;

Model 2 was adjusted for age and gender;

Model 3 was further adjusted for education level (elementary school or below; middle school; high school or above), occupation (coal miners; other blue collars; white collars), household income (<1000 ¥; ≥1000 ¥), smoking status (never; ever; current), alcohol consumption (never; moderate; heavy), physical activity (inactive; occasional; regular), dietary quality (favorable; moderate; unfavorable), antihypertensive drugs (yes or no), lipid-lowering drugs (yes or no), antidiabetic drugs (yes or no), family history of diabetes (yes or no), family history of stroke (yes or no), family history of myocardial infarction (yes or no), and mean of body mass index, high-sensitivity C-reactive protein during 2006 to 2010.

**Table S13** Metabolic syndrome parameters’ variability with risk of stroke among patients after multiple imputation of missing data.

| **Outcome** | **Variable** | **Case/Total, N** | **Follow-up duration**  **(Person-years)** | **Incidence rate, per 1000 person-years** |  | **HR (95%CI)** |  |
| --- | --- | --- | --- | --- | --- | --- | --- |
|  |  |  |  |  | **Model1** | **Model2** | **Model3** |
| **Total stroke** |  | | | | | | |
|  | **0** | 265/4403 | 41544.70 | 3.379 | 1.0 (Reference) | 1.0 (Reference) | 1.0 (Reference) |
|  | **1** | 471/6867 | 64021.07 | 7.357 | 1.16 (1.00, 1.35) | 1.15 (0.99, 1.34) | 1.15 (0.99, 1.35) |
|  | **2** | 328/4559 | 42294.10 | 7.755 | **1.22 (1.04, 1.44)** | **1.22 (1.04, 1.44)** | 1.18 (1.00, 1.40) |
|  | **≥3** | 159/1960 | 17845.74 | 8.910 | **1.41 (1.16, 1.72)** | **1.42 (1.16, 1.73)** | **1.34 (1.09, 1.64)** |
|  | ***P* for trend** | | | | **<0.001** | **<0.001** | **0.005** |
|  | **1 point increment** | | | | **1.11 (1.05, 1.18)** | **1.11 (1.05, 1.18)** | **1.09 (1.02, 1.16)** |
| **Hemorrhagic stroke** |  | | | | | | |
|  | **0** | 36/4403 | 42347.88 | 0.850 | 1.0 (Reference) | 1.0 (Reference) | s1.0 (Reference) |
|  | **1** | 59/6867 | 65429.17 | 0.902 | 1.06 (0.70, 1.61) | 1.06 (0.70, 1.60) | 1.07 (0.69, 1.65) |
|  | **2** | 48/4559 | 43249.17 | 1.110 | 1.31 (0.85, 2.02) | 1.31 (0.85, 2.02) | 1.17 (0.74, 1.86) |
|  | **≥3** | 25/1960 | 18287.41 | 1.367 | 1.62 (0.97, 2.70) | 1.62 (0.97, 2.70) | 1.56 (0.91, 2.67) |
|  | ***P* for trend** | | | | **0.035** | **0.034** | 0.095 |
|  | **1 point increment** | | | | **1.18 (1.01, 1.38)** | **1.18 (1.01, 1.38)** | 1.14 (0.97, 1.35) |
| **Ischemic stroke** |  | | | | | | |
|  | **0** | 239/4403 | 41620.36 | 5.742 | 1.0 (Reference) | 1.0 (Reference) | 1.0 (Reference) |
|  | **1** | 424/6867 | 64161.28 | 6.608 | 1.15 (0.99, 1.35) | 1.15 (0.98, 1.35) | 1.15 (0.98, 1.36) |
|  | **2** | 292/4559 | 42407.76 | 6.886 | **1.21 (1.02, 1.43)** | **1.21 (1.02, 1.43)** | 1.18 (0.99, 1.41) |
|  | **≥3** | 140/1960 | 17899.29 | 7.822 | **1.38 (1.12, 1.70)** | **1.38 (1.12, 1.70)** | **1.31 (1.05, 1.63)** |
|  | ***P* for trend** | | | | **0.002** | **0.002** | **0.013** |
|  | **1 point increment** | | | | **1.10 (1.03, 1.17)** | **1.10 (1.04, 1.17)** | **1.08 (1.02, 1.16)** |

Model 1 was non-adjusted;

Model 2 was adjusted for age and gender;

Model 3 was further adjusted for education level (elementary school or below; middle school; high school or above), occupation (coal miners; other blue collars; white collars), household income (<1000 ¥; ≥1000 ¥), smoking status (never; ever; current), alcohol consumption (never; moderate; heavy), physical activity (inactive; occasional; regular), dietary quality (favorable; moderate; unfavorable), antihypertensive drugs (yes or no), lipid-lowering drugs (yes or no), antidiabetic drugs (yes or no), family history of diabetes (yes or no), family history of stroke (yes or no), family history of myocardial infarction (yes or no), and mean of body mass index, high-sensitivity C-reactive protein during 2006 to 2010.

**Table S14** Associations of variability with risk of total stroke: competing risk analysis.

| **Variability measures** | **Variable** |  | **HR (95%CI)** |  |
| --- | --- | --- | --- | --- |
|  |  | **Model 1** | **Model 2** | **Model 3** |
| **CV** |  | | | |
|  | **0** | 1.0 (Reference) | 1.0 (Reference) | 1.0 (Reference) |
|  | **1** | 1.14 (0.98, 1.33) | 1.13 (0.97, 1.31) | 1.13 (0.97, 1.32) |
|  | **2** | **1.20 (1.02, 1.41)** | **1.19 (1.01, 1.40)** | 1.15 (0.97, 1.36) |
|  | **≥3** | **1.36 (1.12, 1.66)** | **1.35 (1.11, 1.64)** | **1.28 (1.04, 1.58)** |
|  | ***P* for trend** | **0.002** | **0.002** | **0.018** |
|  | **1 point increment** | **1.10 (1.04, 1.16)** | **1.10 (1.03, 1.16)** | **1.08 (1.01, 1.14)** |
| **SD** |  | | | |
|  | **0** | 1.0 (Reference) | 1.0 (Reference) | 1.0 (Reference) |
|  | **1** | 1.10 (0.95, 1.29) | 1.08 (0.93, 1.26) | 1.09 (0.93, 1.28) |
|  | **2** | **1.38 (1.18, 1.62)** | **1.35 (1.15, 1.59)** | **1.27 (1.08, 1.50)** |
|  | **≥3** | **1.47 (1.21, 1.79)** | **1.46 (1.20, 1.78)** | **1.33 (1.08, 1.64)** |
|  | ***P* for trend** | **<0.001** | **<0.001** | **<0.001** |
|  | **1 point increment** | **1.16 (1.09, 1.23)** | **1.15 (1.09, 1.22)** | **1.11 (1.05, 1.18)** |
| **ARV** |  | | | |
|  | **0** | 1.0 (Reference) | 1.0 (Reference) | 1.0 (Reference) |
|  | **1** | 1.10 (0.95, 1.28) | 1.08 (0.93, 1.26) | 1.05 (0.90, 1.23) |
|  | **2** | **1.32 (1.13, 1.55)** | **1.30 (1.11, 1.52)** | **1.21 (1.02, 1.42)** |
|  | **≥3** | **1.40 (1.15, 1.71)** | **1.37 (1.12, 1.66)** | 1.23 (1.00, 1.51) |
|  | ***P* for trend** | **<0.001** | **<0.001** | **<0.001** |
|  | **1 point increment** | **1.13 (1.07, 1.20)** | **1.13 (1.06, 1.19)** | **1.09 (1.02, 1.15)** |
| **VIM** |  | | | |
|  | **0** | 1.0 (Reference) | 1.0 (Reference) | 1.0 (Reference) |
|  | **1** | 1.00 (0.86, 1.15) | 0.98 (0.84, 1.13) | 1.01 (0.87, 1.18) |
|  | **2** | 1.05 (0.90, 1.23) | 1.03 (0.88, 1.21) | 1.04 (0.88, 1.23) |
|  | **≥3** | 1.09 (0.89, 1.33) | 1.06 (0.86, 1.29) | 1.10 (0.89, 1.36) |
|  | ***P* for trend** | 0.278 | 0.442 | 0.311 |
|  | **1 point increment** | 1.03 (0.97, 1.10) | 1.02 (0.96, 1.09) | 1.03 (0.97, 1.10) |

Abbreviations: CV, coefficient of variation; SD, standard deviation; ARV, average real variability; VIM, variability independent of the mean.

Model 1 was non-adjusted;

Model 2 was adjusted for age and gender;

Model 3 was further adjusted for education level (elementary school or below; middle school; high school or above), occupation (coal miners; other blue collars; white collars), household income (<1000 ¥; ≥1000 ¥), smoking status (never; ever; current), alcohol consumption (never; moderate; heavy), physical activity (inactive; occasional; regular), dietary quality (favorable; moderate; unfavorable), antihypertensive drugs (yes or no), lipid-lowering drugs (yes or no), antidiabetic drugs (yes or no), family history of diabetes (yes or no), family history of stroke (yes or no), family history of myocardial infarction (yes or no), and mean of body mass index, high-sensitivity C-reactive protein during 2006 to 2010.

**Table S15** The E-value for metabolic syndrome parameters’ variability with risk of stroke among patients with hypertension.

| **Outcome** | **Exposure** | **HR (95%CI)** | **E-value** | |
| --- | --- | --- | --- | --- |
| **Total stroke** |  |  |  |  |
|  | **0** | 1.0 (Reference) | **-** | **-** |
|  | **1** | 1.15 (0.99, 1.35) | <0.64 | >1.57 |
|  | **2** | 1.18 (1.00, 1.40) | <0.61 | >1.64 |
|  | **≥3** | **1.34 (1.09, 1.64)** | <0.50 | >2.01 |
| **Hemorrhagic stroke** |  |  |  |  |
|  | **0** | 1.0 (Reference) | - | - |
|  | **1** | 1.07 (0.69, 1.65) | <0.75 | >1.34 |
|  | **2** | 1.17 (0.74, 1.86) | <0.62 | >1.62 |
|  | **≥3** | 1.56 (0.91, 2.67) | <0.40 | >2.49 |
| **Ischemic stroke** |  |  |  |  |
|  | **0** | 1.0 (Reference) | - | - |
|  | **1** | 1.15 (0.98, 1.36) | <0.64 | >1.57 |
|  | **2** | 1.18 (0.99, 1.41) | <0.61 | >1.64 |
|  | **≥3** | **1.31 (1.05, 1.63)** | <0.51 | >1.95 |

**Table S16** Baseline characteristics between participants who were excluded and included.

| **Characteristic** | **Not included**  **(N=23185)** | **Included**  **(N=17789)** | ***P*-value** |
| --- | --- | --- | --- |
| **Age, years** | 57.11±11.57 | 52.45±10.92 | **<0.001** |
| **Gender %^*^** |  |  | **<0.001** |
| Women | 3351 (14.45) | 2963 (16.66) |  |
| Men | 19834 (85.55) | 14826 (83.34) |  |
| **Household income per month, %^*^** |  |  | **<0.001** |
| <1000 | 16161 (73.00) | 13137 (74.70) |  |
| ≥1000 | 5977 (27.00) | 4450 (25.30) |  |
| **Education, %^*^** |  |  | **<0.001** |
| Elementary school or below | 801 (9.77) | 1670 (9.40) |  |
| Middle school | 5489 (66.97) | 13008 (73.23) |  |
| High school or further | 1906 (23.26) | 3086 (17.37) |  |
| **Occupation, %^*^** |  |  | **0.019** |
| Coal miners | 6451 (29.79) | 5273 (30.57) |  |
| Blue collars | 13803 (63.74) | 10966 (63.58) |  |
| White collars | 1400 (6.47) | 1008 (5.84) |  |
| **Alcohol drinking, %^*^** |  |  | **<0.001** |
| Never | 13222 (60.11) | 10475 (60.74) |  |
| Moderate | 1073 (4.88) | 594 (3.44) |  |
| Heavy | 7703 (35.02) | 6177 (35.82) |  |
| **Smoking status, %^*^** |  |  | **<0.001** |
| Never | 12684 (56.15) | 11163 (62.80) |  |
| Ever | 1731 (7.66) | 835 (4.70) |  |
| Current | 8176 (36.19) | 5777 (32.50) |  |
| **Dietary quality, %^*^** |  |  | **<0.001** |
| Favorable | 1251 (5.57) | 3230 (18.17) |  |
| Moderate | 17911 (79.68) | 12645 (71.15) |  |
| Unfavorable | 3316 (14.75) | 1898 (10.68) |  |
| **Physical activity, %**^*^ |  |  | **<0.001** |
| Inactive | 1127 (5.02) | 5015 (28.21) |  |
| Occasional | 15731 (70.02) | 10065 (56.62) |  |
| Regular | 5608 (24.96) | 2696 (15.17) |  |
| **Family history of diabetes, %^*^** |  |  | **<0.001** |
| No | 22211 (95.80) | 17175 (97.01) |  |
| Yes | 974 (4.20) | 530 (2.99) |  |
| **Family history of stroke, %^*^** |  |  | 0.069 |
| No | 22217 (95.84) | 16983 (95.47) |  |
| Yes | 965 (4.16) | 806 (4.53) |  |
| **Family history of MI, %^*^** |  |  | **0.011** |
| No | 22788 (98.30) | 17426 (97.96) |  |
| Yes | 394 (1.70) | 363 (2.04) |  |
| **Antihypertensive drugs, %^*^** |  |  | **<0.001** |
| No | 21196 (91.42) | 14108 (79.31) |  |
| Yes | 1989 (8.58) | 3681 (20.69) |  |
| **Lipid-lowering drugs, %^*^** |  |  | 0.216 |
| No | 21909 (98.33) | 17519 (98.48) |  |
| Yes | 373 (1.67) | 270 (1.52) |  |
| **Antidiabetic drugs, %^*^** |  |  | 0.660 |
| No | 22133 (95.46) | 16998 (95.55) |  |
| Yes | 1052 (4.54) | 791 (4.45) |  |
| **WC, cm^†^** | 89.64±9.65 | 89.07±9.56 | **<0.001** |
| **BMI, kg/m^2†^** | 25.83±3.54 | 26.09±3.43 | **<0.001** |
| **SBP, mmHg^†^** | 148.88±17.82 | 146.07±16.83 | **<0.001** |
| **DBP, mmHg^†^** | 92.33±10.72 | 92.70±9.72 | **0.004** |
| **FBG, mmol/L^†^** | 5.70±1.88 | 5.61±1.75 | **<0.001** |
| **HDL-C, mmol/L^†^** | 1.59±0.42 | 1.58±0.41 | 0.1628 |
| **LDL-C, mmol/L^†^** | 2.44±0.94 | 2.36±1.00 | **<0.001** |
| **TG, mmol/L^‡^** | 1.40 (1.00, 2.12) | 1.45 (1.05, 2.26) | **<0.001** |
| **TC, mmol/L^‡^** | 5.02 (4.35, 5.70) | 5.00 (4.35, 5.69) | 0.0826 |
| **Hs-CRP, mg/L^‡^** | 1.00 (0.40, 2.50) | 0.90 (0.33, 2.70) | **<0.001** |

Abbreviations: MI: myocardial infarction, WC: waist circumference, BMI: body mass index, SBP: systolic blood pressure, DBP: diastolic blood pressure, FBG: fasting blood glucose, HDL-C: high-density lipoprotein cholesterol, LDL-C: low-density lipoprotein cholesterol, TG: triglycerides, TC: total cholesterol, Hs-CRP, high-sensitivity C-reactive protein.

**^*^** These variables were examined by χ^2^ tests with H_0_.

**^†^** These variables were examined by 1-way ANOVA with H_0_.

**^‡^** These variables were examined by Mann-Whitney U tests with H_0_. H_0_: There was no statistical difference between the tested variables in included and not included participants (*P*>0.05).

**Table S17** Hazard ratios (HRs) and 95% confidence intervals (95%CIs) of incidence stroke of metabolic parameter variability according to the CV after adjusted for metabolic parameters.

| **Variable** | **Case/Total, N** | **Follow-up duration**  **(Person-years)** | **Incidence rate, per 1000 person-years** | **HR (95%CI)** | | | | | |
| --- | --- | --- | --- | --- | --- | --- | --- | --- | --- |
|  |  |  |  | **Model 1** | **Model 2** | **Model 3** | **Model 4** | **Model 5** | **Model 6** |
| **Total stroke** | | | | | | | | | |
| **0** | 265/4403 | 41544.70 | 6.379 | 1.0 (Reference) | 1.0 (Reference) | 1.0 (Reference) | 1.0 (Reference) | 1.0 (Reference) | 1.0 (Reference) |
| **1** | 471/6867 | 64021.07 | 7.357 | 1.16 (0.99, 1.35) | 1.15 (0.99, 1.34) | 1.15 (0.99, 1.35) | 1.14 (0.97, 1.33) | 1.11 (0.95, 1.29) | 1.12 (0.96, 1.31) |
| **2** | 328/4559 | 42294.10 | 7.755 | **1.22 (1.04, 1.44)** | **1.22 (1.04, 1.44)** | 1.18 (1.00, 1.40) | 1.17 (0.99, 1.38) | 1.10 (0.93, 1.30) | 1.12 (0.95, 1.33) |
| **≥3** | 159/1960 | 17845.74 | 8.910 | **1.41 (1.16, 1.72)** | **1.42 (1.16, 1.73)** | **1.34 (1.09, 1.64)** | **1.32 (1.08, 1.63)** | 1.18 (0.95, 1.45) | **1.24 (1.01, 1.53)** |
| ***P* for trend** | | | | **<0.001** | **<0.001** | **0.005** | **0.009** | 0.170 | 0.057 |
| **1 point increment** | | | | **1.11 (1.05, 1.18)** | **1.11 (1.05, 1.18)** | **1.09 (1.02, 1.16)** | **1.09 (1.02, 1.15)** | 1.05 (0.98, 1.11) | 1.06 (0.999, 1.13) |
| **Hemorrhagic stroke** | | | | | | | | | |
| **0** | 36/4403 | 42347.88 | 0.850 | 1.0 (Reference) | 1.0 (Reference) | 1.0 (Reference) | 1.0 (Reference) | 1.0 (Reference) | 1.0 (Reference) |
| **1** | 59/6867 | 65429.17 | 0.902 | 1.06 (0.70, 1.61) | 1.06 (0.70, 1.60) | 1.07 (0.69, 1.65) | 1.03 (0.67, 1.58) | 1.02 (0.67, 1.57) | 1.04 (0.68, 1.60) |
| **2** | 48/4559 | 43249.17 | 1.110 | 1.31 (0.85, 2.02) | 1.31 (0.85, 2.02) | 1.17 (0.74, 1.86) | 1.13 (0.72, 1.79) | 1.13 (0.71, 1.79) | 1.16 (0.73, 1.84) |
| **≥3** | 25/1960 | 18287.41 | 1.367 | 1.62 (0.97, 2.70) | 1.62 (0.97, 2.70) | 1.56 (0.91, 2.67) | 1.51 (0.88, 2.57) | 1.48 (0.85, 2.56) | 1.59 (0.93, 2.73) |
| ***P* for trend** | | | | **0.035** | **0.034** | 0.095 | 0.130 | 0.159 | 0.090 |
| **1 point increment** | | | | **1.18 (1.01, 1.38)** | **1.18 (1.01, 1.38)** | 1.14 (0.97, 1.35) | 1.13 (0.96, 1.34) | 1.12 (0.95, 1.34) | 1.15 (0.97, 1.36) |
| **Ischemic stroke** | | | | | | | | | |
| **0** | 239/4403 | 41620.36 | 5.742 | 1.0 (Reference) | 1.0 (Reference) | 1.0 (Reference) | 1.0 (Reference) | 1.0 (Reference) | 1.0 (Reference) |
| **1** | 424/6867 | 64161.28 | 6.608 | 1.15 (0.99, 1.35) | 1.15 (0.98, 1.35) | 1.15 (0.98, 1.36) | 1.14 (0.97, 1.34) | 1.11 (0.94, 1.30) | 1.12 (0.95, 1.32) |
| **2** | 292/4559 | 42407.76 | 6.886 | **1.21 (1.02, 1.43)** | **1.21 (1.02, 1.43)** | 1.18 (0.99, 1.41) | 1.17 (0.98, 1.40) | 1.09 (0.92, 1.31) | 1.12 (0.93, 1.33) |
| **≥3** | 140/1960 | 17899.29 | 7.822 | **1.38 (1.12, 1.70)** | **1.38 (1.12, 1.70)** | **1.31 (1.05, 1.63)** | **1.30 (1.05, 1.62)** | 1.14 (0.91, 1.42) | 1.20 (0.97, 1.50) |
| ***P* for trend** | | | | **0.002** | **0.002** | **0.013** | **0.018** | 0.298 | 0.125 |
| **1 point increment** | | | | **1.10 (1.03, 1.17)** | **1.10 (1.04, 1.17)** | **1.08 (1.02, 1.16)** | **1.08 (1.01, 1.15)** | 1.04 (0.97, 1.11) | 1.05 (0.99, 1.13) |

Model 1 was non-adjusted;

Model 2 was adjusted for age and gender;

Model 3 was further adjusted for education level (elementary school or below; middle school; high school or above), occupation (coal miners; other blue collars; white collars), household income (<1000 ¥; ≥1000 ¥), smoking status (never; ever; current), alcohol consumption (never; moderate; heavy), physical activity (inactive; occasional; regular), dietary quality (favorable; moderate; unfavorable), antihypertensive drugs (yes or no), lipid-lowering drugs (yes or no), antidiabetic drugs (yes or no), family history of diabetes (yes or no), family history of stroke (yes or no), family history of myocardial infarction (yes or no), and mean of body mass index, high-sensitivity C-reactive protein during 2006 to 2010.

Model 4: Model3 without adjusted for mean of body mass index, high-sensitivity C-reactive protein during 2006 to 2010.

Model 5: Model3+ mean of waist circumference, systolic blood pressure, fasting blood glucose, high-density lipoprotein cholesterol, and triglycerides during 2006 to 2010.

Model 6: Model3+ baseline waist circumference, systolic blood pressure, fasting blood glucose, high-density lipoprotein cholesterol, and triglycerides**.**

**Table S18** Hazard ratios (HRs) and 95% confidence intervals (95%CIs) of incidence stroke of metabolic parameter variability according to the SD after adjusted for metabolic parameters.

| **Variable** | **Case/Total, N** | **Follow-up duration**  **(Person-years)** | **Incidence rate, per 1000 person-years** | **HR (95%CI)** | | | | | |
| --- | --- | --- | --- | --- | --- | --- | --- | --- | --- |
|  |  |  |  | **Model 1** | **Model 2** | **Model 3** | **Model 4** | **Model 5** | **Model 6** |
| **Total stroke** | | | | | | | | | |
| **0** | 260/4477 | 42366.18 | 6.137 | 1.0 (Reference) | 1.0 (Reference) | 1.0 (Reference) | 1.0 (Reference) | 1.0 (Reference) | 1.0 (Reference) |
| **1** | 435/6798 | 63612.13 | 6.838 | 1.12 (0.96, 1.30) | 1.10 (0.95, 1.29) | 1.11 (0.94, 1.30) | 1.10 (0.93, 1.28) | 1.05 (0.89, 1.23) | 1.07 (0.91, 1.25) |
| **2** | 361/4539 | 41818.63 | 8.633 | **1.42 (1.21, 1.67)** | **1.41 (1.20, 1.65)** | **1.32 (1.12, 1.56)** | **1.31 (1.12, 1.56)** | 1.17 (0.98, 1.39) | **1.22 (1.03, 1.45)** |
| **≥3** | 167/1975 | 17908.67 | 9.325 | **1.54 (1.27, 1.87)** | **1.55 (1.27, 1.88)** | **1.39 (1.13, 1.71)** | **1.40 (1.14, 1.72)** | 1.13 (0.90, 1.41) | 1.23 (1.00, 1.52) |
| ***P* for trend** | | | | **<0.001** | **<0.001** | **<0.001** | **<0.001** | 0.113 | **0.012** |
| **1 point increment** | | | | **1.17 (1.11, 1.24)** | **1.18 (1.11, 1.25)** | **1.13 (1.06, 1.20)** | **1.13 (1.07, 1.21)** | 1.06 (0.99, 1.13) | **1.09 (1.02, 1.16)** |
| **Hemorrhagic stroke** | | | | | | | | | |
| **0** | 37/4477 | 43173.09 | 0.857 | 1.0 (Reference) | 1.0 (Reference) | 1.0 (Reference) | 1.0 (Reference) | 1.0 (Reference) | 1.0 (Reference) |
| **1** | 56/6798 | 64929.70 | 0.863 | 1.01 (0.67, 1.53) | 1.00 (0.66, 1.51) | 1.05 (0.69, 1.62) | 1.02 (0.67, 1.56) | 1.01 (0.66, 1.55) | 1.04 (0.68, 1.60) |
| **2** | 50/4539 | 42839.07 | 1.167 | 1.37 (0.89, 2.09) | 1.35 (0.89, 2.07) | 1.11 (0.70, 1.77) | 1.09 (0.69, 1.72) | 1.07 (0.66, 1.72) | 1.14 (0.71, 1.81) |
| **≥3** | 25/1975 | 18371.78 | 1.361 | 1.60 (0.96, 2.65) | 1.60 (0.96, 2.66) | 1.50 (0.88, 2.57) | 1.47 (0.86, 2.51) | 1.42 (0.80, 2.54) | 1.61 (0.92, 2.80) |
| ***P* for trend** | | | | **0.024** | **0.023** | 0.154 | 0.180 | 0.273 | 0.113 |
| **1 point increment** | | | | **1.19 (1.02, 1.40)** | **1.19 (1.02, 1.40)** | 1.12 (0.95, 1.33) | 1.12 (0.94, 1.32) | 1.10 (0.92, 1.32) | 1.15 (0.96, 1.37) |
| **Ischemic stroke** | | | | | | | | | |
| **0** | 233/4477 | 42442.85 | 5.490 | 1.0 (Reference) | 1.0 (Reference) | 1.0 (Reference) | 1.0 (Reference) | 1.0 (Reference) | 1.0 (Reference) |
| **1** | 393/6798 | 63746.35 | 6.165 | 1.13 (0.96, 1.32) | 1.11 (0.95, 1.31) | 1.11 (0.94, 1.31) | 1.10 (0.93, 1.30) | 1.04 (0.88, 1.24) | 1.06 (0.90, 1.26) |
| **2** | 321/4539 | 41937.31 | 7.654 | **1.41 (1.19, 1.67)** | **1.40 (1.18, 1.65)** | **1.36 (1.12, 1.59)** | **1.34 (1.12, 1.60)** | 1.17 (0.98, 1.41) | **1.23 (1.03, 1.47)** |
| **≥3** | 148/1975 | 17962.19 | 8.240 | **1.52 (1.24, 1.87)** | **1.53 (1.25, 1.88)** | **1.37 (1.10, 1.70)** | **1.39 (1.12, 1.73)** | 1.09 (0.86, 1.37) | 1.19 (0.95, 1.49) |
| ***P* for trend** | | | | **<0.001** | **<0.001** | **<0.001** | **<0.001** | 0.201 | **0.030** |
| **1 point increment** | | | | **1.17 (1.10, 1.24)** | **1.17 (1.10, 1.24)** | **1.13 (1.06, 1.20)** | 1.13 (1.06, 1.21) | 1.05 (0.98, 1.13) | **1.08 (1.01, 1.15)** |

Model 1 was non-adjusted;

Model 2 was adjusted for age and gender;

Model 3 was further adjusted for education level (elementary school or below; middle school; high school or above), occupation (coal miners; other blue collars; white collars), household income (<1000 ¥; ≥1000 ¥), smoking status (never; ever; current), alcohol consumption (never; moderate; heavy), physical activity (inactive; occasional; regular), dietary quality (favorable; moderate; unfavorable), antihypertensive drugs (yes or no), lipid-lowering drugs (yes or no), antidiabetic drugs (yes or no), family history of diabetes (yes or no), family history of stroke (yes or no), family history of myocardial infarction (yes or no), and mean of body mass index, high-sensitivity C-reactive protein during 2006 to 2010.

Model 4: Model3 without adjusted for mean of body mass index, high-sensitivity C-reactive protein during 2006 to 2010.

Model 5: Model3+ mean of waist circumference, systolic blood pressure, fasting blood glucose, high-density lipoprotein cholesterol, and triglycerides during 2006 to 2010.

Model 6: Model3+ baseline waist circumference, systolic blood pressure, fasting blood glucose, high-density lipoprotein cholesterol, and triglycerides**.**

**Table S19** Hazard ratios (HRs) and 95% confidence intervals (95%CIs) of incidence stroke of metabolic parameter variability according to the ARV after adjusted for metabolic parameters.

| **Variable** | **Case/Total, N** | **Follow-up duration**  **(Person-years)** | **Incidence rate, per 1000 person-years** | **HR (95%CI)** | | | | | |
| --- | --- | --- | --- | --- | --- | --- | --- | --- | --- |
|  |  |  |  | **Model 1** | **Model 2** | **Model 3** | **Model 4** | **Model 5** | **Model 6** |
| **Total stroke** | | | | | | | | | |
| **0** | 284/4774 | 45242.63 | 6.277 | 1.0 (Reference) | 1.0 (Reference) | 1.0 (Reference) | 1.0 (Reference) | 1.0 (Reference) | 1.0 (Reference) |
| **1** | 444/6784 | 63358.67 | 7.008 | 1.12 (0.97, 1.30) | 1.10 (0.95, 1.28) | 1.07 (0.92, 1.25) | 1.07 (0.92, 1.25) | 1.01 (0.87, 1.18) | 1.04 (0.89, 1.21) |
| **2** | 344/4400 | 40625.89 | 8.468 | **1.36 (1.16, 1.59)** | **1.35 (1.15, 1.58)** | **1.25 (1.06, 1.47)** | **1.25 (1.06, 1.47)** | 1.11 (0.94, 1.32) | 1.16 (0.98, 1.37) |
| **≥3** | 151/1831 | 16478.41 | 9.164 | **1.48 (1.22, 1.80)** | **1.47 (1.20, 1.79)** | **1.30 (1.06, 1.61)** | **1.32 (1.08, 1.63)** | 1.05 (0.84, 1.31) | 1.14 (0.92, 1.42) |
| ***P* for trend** | | | | **<0.001** | **<0.001** | **0.001** | **0.001** | 0.362 | 0.077 |
| **1 point increment** | | | | **1.15 (1.09, 1.22)** | **1.15 (1.09, 1.22)** | **1.11 (1.04, 1.18)** | **1.11 (1.04, 1.18)** | 1.03 (0.97, 1.10) | 1.06 (0.99, 1.13) |
| **Hemorrhagic stroke** | | | | | | | | | |
| **0** | 43/4774 | 46132.51 | 0.932 | 1.0 (Reference) | 1.0 (Reference) | 1.0 (Reference) | 1.0 (Reference) | 1.0 (Reference) | 1.0 (Reference) |
| **1** | 57/6784 | 64626.43 | 0.882 | 0.95 (0.64, 1.41) | 0.94 (0.63, 1.39) | 0.86 (0.57, 1.30) | 0.88 (0.58, 1.32) | 0.87 (0.57, 1.31) | 0.89 (0.59, 1.35) |
| **2** | 43/4400 | 41668.32 | 1.032 | 1.11 (0.73, 1.70) | 1.10 (0.72, 1.68) | 0.95 (0.61, 1.48) | 0.95 (0.61, 1.49) | 0.93 (0.58, 1.48) | 0.98 (0.62, 1.55) |
| **≥3** | 25/1831 | 16886.38 | 1.481 | 1.60 (0.98, 2.62) | 1.58 (0.97, 2.59) | 1.37 (0.81, 2.33) | 1.39 (0.82, 2.36) | 1.32 (0.75, 2.34) | 1.48 (0.85, 2.56) |
| ***P* for trend** | | | | 0.063 | 0.069 | 0.278 | 0.284 | 0.424 | 0.219 |
| **1 point increment** | | | | 1.15 (0.98, 1.35) | 1.15 (0.98, 1.34) | 1.08 (0.91, 1.28) | 1.08 (0.92, 1.29) | 1.06 (0.89, 1.28) | 1.10 (0.92, 1.32) |
| **Ischemic stroke** | | | | | | | | | |
| **0** | 254/4774 | 45330.48 | 5.603 | 1.0 (Reference) | 1.0 (Reference) | 1.0 (Reference) | 1.0 (Reference) | 1.0 (Reference) | 1.0 (Reference) |
| **1** | 399/6784 | 63516.01 | 6.282 | 1.13 (0.96, 1.32) | 1.11 (0.95, 1.30) | 1.08 (0.92, 1.27) | 1.08 (0.92, 1.27) | 1.01 (0.86, 1.20) | 1.09 (0.93, 1.26) |
| **2** | 309/4400 | 40711.54 | 7.590 | **1.36 (1.16, 1.61)** | **1.35 (1.15, 1.60)** | **1.27 (1.07, 1.51)** | **1.27 (1.07, 1.51)** | 1.12 (0.94, 1.34) | 1.15 (0.91, 1.46) |
| **≥3** | 133/1831 | 16530.66 | 8.046 | **1.46 (1.18, 1.80)** | **1.44 (1.17, 1.78)** | **1.30 (1.04, 1.62)** | **1.32 (1.06, 1.64)** | 1.02 (0.80, 1.29) | 0.92 (0.58, 1.47) |
| ***P* for trend** | | | | **<0.001** | **<0.001** | **0.002** | **0.001** | 0.478 | 0.126 |
| **1 point increment** | | | | **1.15 (1.08, 1.22)** | **1.15 (1.08, 1.22)** | **1.11 (1.04, 1.18)** | **1.11 (1.04, 1.19)** | 1.03 (0.96, 1.10) | 1.06 (0.99, 1.13) |

Model 1 was non-adjusted;

Model 2 was adjusted for age and gender;

Model 3 was further adjusted for education level (elementary school or below; middle school; high school or above), occupation (coal miners; other blue collars; white collars), household income (<1000 ¥; ≥1000 ¥), smoking status (never; ever; current), alcohol consumption (never; moderate; heavy), physical activity (inactive; occasional; regular), dietary quality (favorable; moderate; unfavorable), antihypertensive drugs (yes or no), lipid-lowering drugs (yes or no), antidiabetic drugs (yes or no), family history of diabetes (yes or no), family history of stroke (yes or no), family history of myocardial infarction (yes or no), and mean of body mass index, high-sensitivity C-reactive protein during 2006 to 2010.

Model 4: Model3 without adjusted for mean of body mass index, high-sensitivity C-reactive protein during 2006 to 2010.

Model 5: Model3+ mean of waist circumference, systolic blood pressure, fasting blood glucose, high-density lipoprotein cholesterol, and triglycerides during 2006 to 2010.

Model 6: Model3+ baseline waist circumference, systolic blood pressure, fasting blood glucose, high-density lipoprotein cholesterol, and triglycerides**.**

**Table S20** Hazard ratios (HRs) and 95% confidence intervals (95%CIs) of incidence stroke of metabolic parameter variability according to the VIM after adjusted for metabolic parameters.

| **Variable** | **Case/Total, N** | **Follow-up duration**  **(Person-years)** | **Incidence rate, per 1000 person-years** | **HR (95%CI)** | | | | | |
| --- | --- | --- | --- | --- | --- | --- | --- | --- | --- |
|  |  |  |  | **Model 1** | **Model 2** | **Model 3** | **Model 4** | **Model 5** | **Model 6** |
| **Total stroke** | | | | | | | | | |
| **0** | 293/4354 | 40984.24 | 7.149 | 1.0 (Reference) | 1.0 (Reference) | 1.0 (Reference) | 1.0 (Reference) | 1.0 (Reference) | 1.0 (Reference) |
| **1** | 465/6934 | 64800.44 | 7.176 | 1.01 (0.87, 1.16) | 1.00 (0.86, 1.16) | 1.03 (0.89, 1.20) | 1.02 (0.88, 1.18) | 1.01 (0.87, 1.18) | 1.01 (0.87, 1.17) |
| **2** | 324/4578 | 42370.73 | 7.647 | 1.07 (0.92, 1.26) | 1.07 (0.91, 1.25) | 1.08 (0.91, 1.27) | 1.06 (0.90, 1.24) | 1.06 (0.90, 1.25) | 1.06 (0.90, 1.25) |
| **≥3** | 141/1923 | 17550.20 | 8.034 | 1.13 (0.93, 1.39) | 1.13 (0.92, 1.38) | 1.17 (0.95, 1.44) | 1.14 (0.92, 1.40) | 1.13 (0.92, 1.40) | 1.14 (0.92, 1.40) |
| ***P* for trend** | | | | 0.141 | 0.160 | 0.110 | 0.199 | 0.196 | 0.176 |
| **1 point increment** | | | | 1.04 (0.98, 1.11) | 1.04 (0.98, 1.11) | 1.05 (0.99, 1.12) | 1.03 (0.98, 1.11) | 1.04 (0.98, 1.11) | 1.04 (0.98, 1.11) |
| **Hemorrhagic stroke** | | | | | | | | | |
| **0** | 39/4354 | 41816.87 | 0.932 | 1.0 (Reference) | 1.0 (Reference) | 1.0 (Reference) | 1.0 (Reference) | 1.0 (Reference) | 1.0 (Reference) |
| **1** | 56/6934 | 66186.66 | 0.846 | 0.91 (0.60, 1.37) | 0.91 (0.60, 1.36) | 0.95 (0.62, 1.46) | 0.92 (0.60, 1.40) | 0.91 (0.60, 1.40) | 0.91 (0.60, 1.39) |
| **2** | 52/4578 | 43341.65 | 1.120 | 1.29 (0.85, 1.96) | 1.29 (0.85, 1.95) | 1.24 (0.79, 1.93) | 1.19 (0.77, 1.85) | 1.20 (0.77, 1.86) | 1.20 (0.77, 1.86) |
| **≥3** | 21/1923 | 17938.17 | 1.171 | 1.26 (0.74, 2.15) | 1.26 (0.74, 2.14) | 1.21 (0.68, 2.14) | 1.16 (0.66, 2.04) | 1.16 (0.66, 2.05) | 1.17 (0.67, 2.07) |
| ***P* for trend** | | | | 0.120 | 0.123 | 0.245 | 0.318 | 0.309 | 0.288 |
| **1 point increment** | | | | 1.13 (0.96, 1.32) | 1.13 (0.96, 1.32) | 1.10 (0.93, 1.30) | 1.09 (0.92, 1.29) | 1.09 (0.92, 1.29) | 1.09 (0.92, 1.29) |
| **Ischemic stroke** | | | | | | | | | |
| **0** | 266/4354 | 41023.93 | 6.479 | 1.0 (Reference) | 1.0 (Reference) | 1.0 (Reference) | 1.0 (Reference) | 1.0 (Reference) | 1.0 (Reference) |
| **1** | 420/6934 | 64936.69 | 6.468 | 1.00 (0.86, 1.17) | 0.99 (0.85, 1.16) | 1.02 (0.87, 1.20) | 1.01 (0.86, 1.19) | 1.01 (0.86, 1.18) | 1.00 (0.85, 1.17) |
| **2** | 283/4578 | 42499.27 | 6.659 | 1.03 (0.87, 1.22) | 1.03 (0.87, 1.21) | 1.04 (0.88, 1.24) | 1.02 (0.86, 1.22) | 1.03 (0.86, 1.22) | 1.03 (0.86, 1.22) |
| **≥3** | 126/1923 | 17598.51 | 7.160 | 1.11 (0.90, 1.38) | 1.11 (0.90, 1.37) | 1.17 (0.94, 1.45) | 1.14 (0.92, 1.42) | 1.13 (0.91, 1.41) | 1.13 (0.91, 1.41) |
| ***P* for trend** | | | | 0.334 | 0.370 | 0.222 | 0.279 | 0.292 | 0.270 |
| **1 point increment** | | | | 1.03 (0.97, 1.09) | 1.03 (0.97, 1.09) | 1.04 (0.98, 1.11) | 1.03 (0.97, 1.10) | 1.03 (0.97, 1.10) | 1.03 (0.97, 1.10) |

Model 1 was non-adjusted;

Model 2 was adjusted for age and gender;

Model 3 was further adjusted for education level (elementary school or below; middle school; high school or above), occupation (coal miners; other blue collars; white collars), household income (<1000 ¥; ≥1000 ¥), smoking status (never; ever; current), alcohol consumption (never; moderate; heavy), physical activity (inactive; occasional; regular), dietary quality (favorable; moderate; unfavorable), antihypertensive drugs (yes or no), lipid-lowering drugs (yes or no), antidiabetic drugs (yes or no), family history of diabetes (yes or no), family history of stroke (yes or no), family history of myocardial infarction (yes or no), and mean of body mass index, high-sensitivity C-reactive protein during 2006 to 2010.

Model 4: Model3 without adjusted for mean of body mass index, high-sensitivity C-reactive protein during 2006 to 2010.

Model 5: Model3+ mean of waist circumference, systolic blood pressure, fasting blood glucose, high-density lipoprotein cholesterol, and triglycerides during 2006 to 2010.

Model 6: Model3+ baseline waist circumference, systolic blood pressure, fasting blood glucose, high-density lipoprotein cholesterol, and triglycerides**.**

**Table S21** Hazard ratios (HRs) and 95% confidence intervals (95%CIs) of total stroke by quartiles of metabolic parameter variability according to the coefficient of variation after adjusted for metabolic parameters.

| **Variable** | **Case/Total, N** | **Follow-up duration**  **(Person-years)** | **Incidence rate, per 1000 person-years** | **HR (95%CI)** | | | | | |
| --- | --- | --- | --- | --- | --- | --- | --- | --- | --- |
|  |  |  |  | **Model 1** | **Model 2** | **Model 3** | **Model 4** | **Model 5** | **Model 6** |
| **Waist Circumstance** | | | | | | | | | |
| Q1 | 309/4448 | 41933.47 | 7.367 | 1.0 (Reference) | 1.0 (Reference) | 1.0 (Reference) | 1.0 (Reference) | 1.0 (Reference) | 1.0 (Reference) |
| Q2 | 293/4450 | 41651.91 | 7.035 | 0.96 (0.81, 1.12) | 0.96 (0.82, 1.13) | 0.95 (0.81, 1.12) | 0.95 (0.81, 1.12) | 0.96 (0.81, 1.13) | 0.95 (0.81, 1.12) |
| Q3 | 317/4445 | 41290.31 | 7.677 | 1.04 (0.89, 1.22) | 1.03 (0.88, 1.20) | 1.03 (0.87, 1.21) | 1.02 (0.87, 1.20) | 1.03 (0.88, 1.21) | 1.02 (0.86, 1.20) |
| Q4 | 304/4446 | 40829.91 | 7.446 | 1.01 (0.87, 1.19) | 0.97 (0.82, 1.13) | 0.95 (0.81, 1.13) | 0.95 (0.80, 1.12) | 0.96 (0.81, 1.13) | 0.93 (0.79, 1.10) |
| ***P* for trend** | | | | 0.650 | 0.817 | 0.791 | 0.654 | 0.736 | 0.498 |
| **1 point increment** | | | | 1.01 (0.96, 1.07) | 1.00 (0.95, 1.05) | 0.99 (0.94, 1.05) | 0.99 (0.94, 1.04) | 0.99 (0.94, 1.05) | 0.98 (0.93, 1.04) |
| **Systolic Blood Pressure** | | | | | | | | | |
| Q1 | 287/4448 | 42032.15 | 6.828 | 1.0 (Reference) | 1.0 (Reference) | 1.0 (Reference) | 1.0 (Reference) | 1.0 (Reference) | 1.0 (Reference) |
| Q2 | 298/4534 | 42408.35 | 7.027 | 1.03 (0.88, 1.21) | 1.02 (0.87, 1.20) | 1.02 (0.86, 1.20) | 1.02 (0.86, 1.21) | 0.99 (0.83, 1.17) | 1.01 (0.85, 1.19) |
| Q3 | 287/4360 | 40654.39 | 7.060 | 1.04 (0.88, 1.22) | 1.01 (0.86, 1.20) | 1.01 (0.85, 1.20) | 1.02 (0.86, 1.20) | 1.00 (0.84, 1.18) | 1.01 (0.86, 1.20) |
| Q4 | 351/4447 | 40610.70 | 8.643 | **1.28 (1.09, 1.49)** | **1.24 (1.06, 1.45)** | **1.24 (1.05, 1.46)** | **1.23 (1.05, 1.45)** | 1.17 (1.00, 1.38) | **1.23 (1.05, 1.45)** |
| ***P* for trend** | | | | **0.001** | **0.005** | **0.005** | **0.007** | **0.031** | **0.006** |
| **1 point increment** | | | | **1.08 (1.03, 1.14)** | **1.07 (1.02, 1.12)** | **1.07 (1.01, 1.13)** | **1.07 (1.01, 1.12)** | 1.05 (1.00, 1.11) | **1.07 (1.01, 1.13)** |
| **Fasting Blood Glucose** | | | | | | | | | |
| Q1 | 271/4448 | 41931.71 | 6.463 | 1.0 (Reference) | 1.0 (Reference) | 1.0 (Reference) | 1.0 (Reference) | 1.0 (Reference) | 1.0 (Reference) |
| Q2 | 316/4447 | 41496.39 | 7.615 | **1.18 (1.005, 1.39)** | 1.14 (0.97, 1.34) | 1.14 (0.96, 1.34) | 1.13 (0.96, 1.33) | 1.12 (0.95, 1.33) | 1.12 (0.95, 1.33) |
| Q3 | 296/4447 | 41429.19 | 7.145 | 1.11 (0.94, 1.31) | 1.06 (0.90, 1.25) | 1.06 (0.89, 1.25) | 1.05 (0.89, 1.24) | 1.03 (0.87, 1.22) | 1.03 (0.87, 1.23) |
| Q4 | 340/4447 | 40848.30 | 8.324 | **1.30 (1.11, 1.52)** | **1.25 (1.07, 1.47)** | 1.13 (0.95, 1.34) | 1.14 (0.96, 1.35) | 1.01 (0.85, 1.21) | 1.06 (0.90, 1.27) |
| ***P* for trend** | | | | **0.004** | **0.013** | 0.316 | 0.264 | 0.751 | 0.768 |
| **1 point increment** | | | | **1.07 (1.02, 1.13)** | **1.06 (1.01, 1.12)** | 1.03 (0.97, 1.08) | 1.03 (0.98, 1.09) | 0.99 (0.94, 1.05) | 1.01 (0.96, 1.07) |
| **High-Density Lipoprotein** | | | | | | | | | |
| Q1 | 286/4448 | 41374.63 | 6.912 | 1.0 (Reference) | 1.0 (Reference) | 1.0 (Reference) | 1.0 (Reference) | 1.0 (Reference) | 1.0 (Reference) |
| Q2 | 288/4447 | 41638.66 | 6.917 | 1.00 (0.85, 1.18) | 1.04 (0.88, 1.22) | 1.10 (0.92, 1.30) | 1.10 (0.93, 1.31) | 1.11 (0.93, 1.32) | 1.11 (0.93, 1.32) |
| Q3 | 308/4447 | 41496.77 | 7.422 | 1.08 (0.92, 1.26) | 1.13 (0.96, 1.33) | **1.20 (1.02, 1.42)** | **1.19 (1.01, 1.41)** | **1.21 (1.02, 1.43)** | **1.20 (1.01, 1.41)** |
| Q4 | 341/4447 | 41195.55 | 8.278 | **1.20 (1.03, 1.41)** | **1.23 (1.05, 1.44)** | **1.29 (1.09, 1.52)** | **1.28 (1.09, 1.52)** | **1.32 (1.12, 1.56)** | **1.29 (1.10, 1.52)** |
| ***P* for trend** | | | | **0.009** | **0.004** | **0.001** | **0.002** | **0.001** | **0.002** |
| **1 point increment** | | | | **1.07 (1.01, 1.12)** | **1.07 (1.02, 1.13)** | **1.09 (1.03, 1.15)** | **1.09 (1.03, 1.14)** | **1.10 (1.04, 1.15)** | **1.09 (1.03, 1.15)** |
| **Triglycerides** | | | | | | | | | |
| Q1 | 317/4448 | 41287.65 | 7.678 | 1.0 (Reference) | 1.0 (Reference) | 1.0 (Reference) | 1.0 (Reference) | 1.0 (Reference) | 1.0 (Reference) |
| Q2 | 314/4447 | 41150.86 | 7.631 | 1.00 (0.85, 1.16) | 1.00 (0.86, 1.17) | 0.98 (0.84, 1.16) | 0.99 (0.84, 1.17) | 0.99 (0.84, 1.16) | 0.99 (0.84, 1.16) |
| Q3 | 291/4447 | 41360.72 | 7.036 | 0.92 (0.78, 1.07) | 0.95 (0.81, 1.11) | 0.93 (0.79, 1.09) | 0.93 (0.79, 1.10) | 0.92 (0.78, 1.09) | 0.93 (0.79, 1.09) |
| Q4 | 301/4447 | 41906.38 | 7.183 | 0.93 (0.80, 1.09) | 1.03 (0.88, 1.21) | 1.01 (0.86, 1.20) | 1.02 (0.86, 1.20) | 0.97 (0.82, 1.15) | 0.99 (0.84, 1.17) |
| ***P* for trend** | | | | 0.306 | 0.798 | 0.879 | 0.870 | 0.612 | 0.849 |
| **1 point increment** | | | | 0.97 (0.92, 1.02) | 1.00 (0.95, 1.05) | 1.00 (0.95, 1.05) | 1.00 (0.95, 1.05) | 0.98 (0.93, 1.04) | 0.99 (0.94, 1.04) |

Model 1 was non-adjusted;

Model 2 was adjusted for age and gender;

Model 3 was further adjusted for education level (elementary school or below; middle school; high school or above), occupation (coal miners; other blue collars; white collars), household income (<1000 ¥; ≥1000 ¥), smoking status (never; ever; current), alcohol consumption (never; moderate; heavy), physical activity (inactive; occasional; regular), dietary quality (favorable; moderate; unfavorable), antihypertensive drugs (yes or no), lipid-lowering drugs (yes or no), antidiabetic drugs (yes or no), family history of diabetes (yes or no), family history of stroke (yes or no), family history of myocardial infarction (yes or no), and mean of body mass index, high-sensitivity C-reactive protein during 2006 to 2010.

Model 4: Model3 without adjusted for mean of body mass index, high-sensitivity C-reactive protein during 2006 to 2010.

Model 5: Model3+ mean of waist circumference, systolic blood pressure, fasting blood glucose, high-density lipoprotein cholesterol, and triglycerides during 2006 to 2010.

Model 6: Model3+ baseline waist circumference, systolic blood pressure, fasting blood glucose, high-density lipoprotein cholesterol, and triglycerides**.**
